# Bioinspired Vascular Bundle Structured Nanocellulose/PVDF-HFP Composite Membranes for Efficient Ion Transport and Stable All-Solid-State Lithium Batteries

**DOI:** 10.1007/s40820-026-02092-0

**Published:** 2026-02-14

**Authors:** Chenxiang Gao, Yijie Zhou, Yun Huang, Shuhui Wang, Xiaoyan Ma

**Affiliations:** 1 School of Chemistry and Chemical Engineering, Northwestern Polytechnical University, Xi’an, 710072 People’s Republic of China; 2 School of New Energy and Materials, Southwest Petroleum University, Chengdu, 610500 People’s Republic of China

**Keywords:** Nanocellulose, PVDF-HFP, Separators, Lithium batteries, Polymer electrolytes

## Abstract

In-situ polymerization of solid-state polymer electrolytes is a promising approach for achieving mass production of all-solid-state batteries. However, inferior ionic conductivity and separator infiltration limit practical applications. Inspired by the nutrient-transporting vascular bundles in plants, a biomimetic fluorinated nanocellulose/PVDF-HFP porous composite membrane of stacked parallel nanocellulose bundles wrapped by PVDF-HFP sheaths is designed and prepared. The nanocellulose bundles assembled of fluorinated cellulose nanocrystals and cellulose nanofibers through shear-induced alignment create low-curvature ion transport channels, while the PVDF-HFP sheath facilitates lithium salt dissociation and reinforces structural stability. Benefiting from this bundle-sheath structure, the composite membrane exhibits excellent ionic conductivity, stability, and electrolyte wettability. The polymer electrolyte prepared with this composite membrane has a high ionic conductivity of 2.46 × 10^−4^ S cm^−1^ (30 °C), an electrochemical stability window (5.3 V), and cycle stability. Consequently, Li||LFP cells can retain a superior capacity of 77.48% after 1000 cycles at 1 C, and Li||NCM811 cells can maintain 83.94% capacity after 300 cycles at 0.1 C. Moreover, pouch cells can withstand temperatures up to 130 °C without thermal runaway. This biomimetic strategy provides a promising pathway to advance cellulose separators for high-performance all-solid-state batteries.

**Supplementary Information:**

The online version contains supplementary material available at 10.1007/s40820-026-02092-0.

## Highlights

- Bioinspired bundle-sheath structure improves electrochemical and thermal stability; nanocellulose composite membranes can be used as high-performance all-solid-state lithium batteries separators.
- The FFP/ASSPE has a high ionic conductivity of 2.46 × 10^−4^ S cm^−1^ at 30 °C.
- Li|FFP/ASSPE|LFP cells can maintain 77.48% capacity after 1000 cycles at 1 C, and Li|FFP/ASSPE|NCM811 cells maintain 83.94% capacity after 300 cycles of 0.1 C.

**Supplementary Information:**

The online version contains supplementary material available at 10.1007/s40820-026-02092-0.

## Graphical abstract

**Figure:**
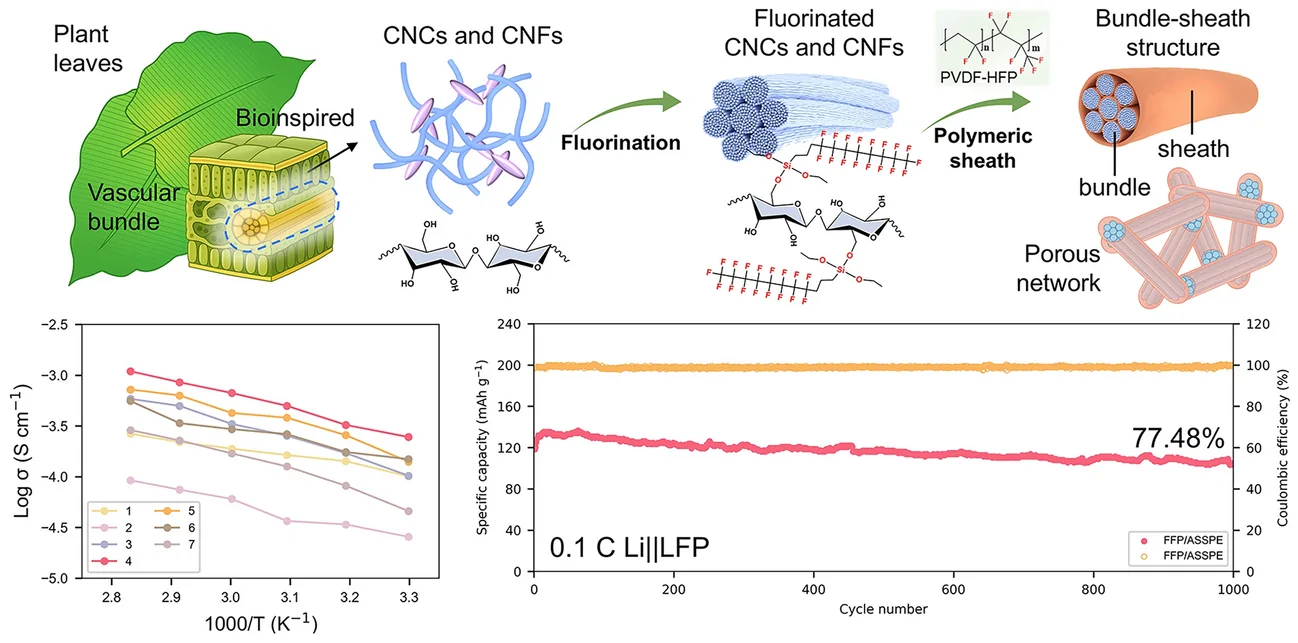

## Introduction

All-solid-state lithium batteries (ASSLBs) offer outstanding safety and energy density, providing solutions for electric vehicles and large-scale grid storage [1–3]. However, their practical deployment is hindered by slow ion transport and unstable solid–solid interfaces [4, 5]. The in-situ polymerization of all-solid-state polymer electrolytes (ASSPEs) with high flexibility and interface stability is an effective method for optimizing solid–solid interfaces and long-term stable cycling of batteries [6, 7]. Currently, developing separators with excellent wettability for in-situ polymerization and addressing the inherently low ionic conductivity of ASSPEs remain critical challenges [8]. Commercial polyolefin separators suffer from low wettability, inferior heat resistance, and limited electrochemical stability [9], making them unsuitable for high-performance ASSLBs. Therefore, the development of novel separators with excellent wettability, high ionic conductivity, and electrochemical stability is imperative.

Cellulose-based separators have attracted increasing attention in rechargeable batteries because of their high thermal stability, excellent electrolyte wettability, ability to suppress the shuttle effect, renewability, and natural abundance [10, 11]. Seo et al. prepared a chiral nematic liquid crystalline cellulose nanocrystal separator with good cycle stability (84% after 100 cycles, Li||NCM811) [12]. Ping et al. fabricated a Cu(OH)_2_ nanocluster-enhanced cellulose composite separator, which achieved high ionic conductivity (2.14 × 10^−3^ S cm^−1^) [13]. These reports demonstrate the application potential of cellulose in rechargeable battery separators. However, the polyhydroxy structure and the insufficient mechanical strength of cellulose result in substandard oxidative stability and cycle stability. To improve these issues, reported strategies typically involve chemical treatment (reducing the hydroxyl density) and composite reinforcement (compositing polymers or inorganic fillers) [14–16]. For example, Wang et al. replaced some hydroxyl groups in cellulose with cyanide-containing structures, enhancing both the electrochemical stability and ionic conductivity (1.25 × 10^−3^ S cm^−1^) [17]. Liu et al. prepared a composite membrane with an outstanding pore structure and exceptional mechanical properties (25.4 MPa) by reinforcing cellulose nanofibers with polyimide [18]. Nevertheless, chemical modification often compromises the ability to form membranes. Composite modification is simple, but the obtained structures are generally disordered, failing to guarantee continuous ion transport channels and resulting in limited ionic conductivity. Therefore, achieving membranes with both mechanically stable and continuous ion transport channels remains a significant challenge.

Vascular bundles are bundle-like structures in vascular plants and are typically surrounded by one or more layers of vascular bundle-sheath cells, together forming a composite bundle-sheath structure [19, 20]. This composite structure is responsible for transporting organic nutrients throughout the plant body while also providing structural support. This function stems from its unique geometry. The abundant voids between vascular bundles provide space for the infiltration of nutrients, while the parallel arrangement of these bundles creates straight channels, offering low-curvature channels for nutrient transport. Additionally, the outer sheath cells provide high mechanical strength. This vascular bundle structure inspires the development of separators with continuous ion transport channels and enhanced stability.

In this paper, we innovatively propose a vascular bundle structure biomimetic strategy for developing fluorinated nanocellulose/PVDF-HFP bundle-sheath structures and high-performance membranes. Specifically, fluorinated cellulose nanofibers with high aspect ratios exhibit a local orientation along the flow direction under a weak shear stress gradient and pull fluorinated cellulose nanocrystals through hydrogen bonding and dipole interactions. This process results in the parallel alignment of the nanocellulose molecular chains. Through the continuous assembly of these two types of fluorinated nanocellulose, parallel molecular chains progressively accumulate, self-assemble into microfibrils, and further assemble into bundles. Moreover, PVDF-HFP segments with identical –CF_2_– are attracted to the nanocellulose bundles by dipole interactions and van der Waals forces, enveloping them to form sheaths. Among these parallel nanocellulose molecular chains, microfibrils, and bundles, numerous low-curvature interstices exist, serving as ion transport channels. PVDF-HFP sheaths promote lithium salt dissociation and contribute to structural stability. This bundle-sheath structure stacks within the membrane, forming a porous network to absorb the polymer precursor. The bundle-sheath structure endows the composite membranes with high tensile strength (11.8 MPa). The all-solid-state polymer electrolytes prepared by in-situ polymerization with these composite membranes as separators have a high ionic conductivity of 2.46 × 10^−4^ S cm^−1^ (30 °C) and a high potential window (5.3 V). The composite membrane endows the polymer electrolyte with outstanding cycling stability and compatibility with high-voltage cathodes. The Li||Li cells can operate stably for more than 1200 h at a current density of 0.2 mA cm^−2^ without short circuits. For Li||LFP cells, a high capacity retention of 77.48% is maintained after 1000 cycles at 1 C, and 86.09% is maintained after 100 cycles at 5 C. The Li||NCM811 cells exhibit a superior capacity retention of 83.94% after 300 cycles at 0.1 C. This strategy is expected to enhance the adaptability of cellulose separators in high-voltage systems and provide new insight for the development of high-performance all-solid-state lithium batteries.

## Experimental Section

### Materials

Nanocelluloses and 1H,1H,2H,2H-perfluorodecyltriethoxysilane (PFDTES, 96%, Rharn) were used to prepare fluorinated nanocelluloses. Sodium hydroxide (NaOH, AR, Rharn) and dilute hydrochloric acid were used to regulate pH. N,N-dimethylformamide (AR, 99.5%, Rharn) and poly(vinylidene fluoride-co-hexafluoropropylene) (PVDF-HFP, average Mw ~ 400,000, iVsci) were used for the preparation of the composite membranes. Vinylene carbonate (VC, 98%, Rharn), 2,2,2-trifluoroethyl methacrylate (TFA, 98%, Rharn), azobisisobutyronitrile (AIBN, ≥ 98%, aladdin), 1-vinyl-3-pentyl cyano imidazole (trifluoromethanesulfonyl) imide (VCIM-TFSI), octamethacryloyloxypropyl polyhedral oligomeric silsesquioxane (octaMA-POSS), and lithium bis((trifluoromethyl)sulfonyl)azanide (LiTFSI, 99%, OriLeaf) were used for in-situ polymerization.

### Preparation of Fluorinated Nanocelluloses, Composite Membranes, and ASSPEs

#### Preparations of the Fluorinated Nanocelluloses

The pH of the anhydrous ethanol/deionized water (30/2 mL) solution was adjusted to 5.5, then PFDTES (4 g) was added and stirred for 1 h, recorded as Solution A. The nanocelluloses (1 g, CNCs or CNFs) were dispersed in an anhydrous ethanol/deionized water (50/2 mL) solution and stirred at 40 °C for 30 min, recorded as Solution B. Solution B was next slowly dripped into Solution A using a funnel for 30 min. The reaction was carried out under a nitrogen atmosphere and at 60 °C for 24 h [21, 22]. The product was washed several times with anhydrous ethanol and dried under vacuum at 80 °C for 24 h to obtain fluorinated nanocelluloses (F-CNC or F-CNF). See Fig. S1 for the structural formula. The preparation of nanocelluloses is described in the Supporting Information.

#### Preparations of the Composite Membranes

Firstly, dissolve a specified amount of PVDF-HFP in N,N-dimethylformamide (DMF) and stir for at least 2 h to ensure complete dissolution. Secondly, add F-CNC and F-CNF (mass ratio 1:3) to the PVDF-HFP solution and stir thoroughly for more than 4 h. Then, place the dispersion in a vacuum to remove bubbles. Finally, apply the dispersion to the polytetrafluoroethylene plate and vacuum dry at 80 °C for 48 h to obtain composite membranes (recorded as FFP, see Table S1 for detailed component ratios). The PVDF-HFP membranes without fluorinated nanocellulose were prepared using the same method. The pure fluorinated nanocellulose membranes (recorded as FF) were also prepared (see the Supporting Information for the preparation method).

#### Preparations of the ASSPEs

Briefly, VC (0.1 g), TFA (0.2 g), VCIM-TFSI (0.1 g), octaMA-POSS (0.0054 g), AIBN (0.0015 g), and LiTFSI (0.0574 g) were mixed thoroughly in a glove box to prepare the electrolyte polymer precursor. Then, the cells were assembled in order, using FFP membranes (FF or PVDF-HFP membranes) as the separators, with 30 µL polymer precursor. Finally, the encapsulated cells were placed in an 80 °C oven for 24 h to cure. The prepared electrolytes were recorded as FFP/ASSPE (FF/ASSPE or PVDF-HPF/ASSPE). The thickness of FFP/ASSPE is about 80 µm. The synthesis of octaMA-POSS and VCIM-TFSI is described in Figs. S2 and S3.

### Materials Characterization

The contact angles of the membranes were tested using a contact angle measuring instrument (DSA100). The scanning electron microscopy (SEM, Verios G4) and transmission electron microscope (TEM, Themis Z) were used to observe the morphology of membranes and electrolytes. The structure of the materials was analyzed by Fourier transform infrared spectroscopy (FT-IR, TENSOR 27). A Raman spectrometer (Alpha300R) was used to study the dissociation of lithium salts. The simultaneous thermal analysis (STA 449F3) and differential scanning calorimeter (PerkinElmer DSC 8000) were used to analyze the thermal properties of membranes and electrolytes. The mechanical properties of membranes and electrolytes were tested by a homemade tensile testing machine. X-ray photoelectron spectroscopy (XPS, Kratos AXIS Ultra DLD) was used to study the elemental information on the surface of lithium electrodes after cycling.

### Electrochemical Characterization

The dielectric constant and dissipation factor of the membranes were measured using a broadband dielectric spectrometer (Concept 80). The ferroelectric tester (FERRO20B) was used to test the curves of the potential shift of the membranes versus the strength of the electric field (D-E Loops). Linear sweep voltammetry (LSV) curves of electrolytes were tested by an electrochemical workstation (CHI660e). Use an Autolab workstation (PGSTAT 302 N) to study the electrochemical impedance spectroscopy and i-t curves of electrolytes. The charging and discharging of the cells were controlled by LAND battery testers (CT3001A). The ionic conductivities (*σ*, S cm^−1^) of electrolytes were calculated based on electrochemical impedance spectroscopy and Eq. 1 [23, 24]:

1 $$\sigma = \frac{L}{{R_{\mathrm{b}} \times S}}$$

where *L* (cm) is the thickness of electrolyte, *R*_*b*_ (Ω) is the bulk resistance of the electrolyte, and *S* (cm^2^) is the effective contact area between electrodes/electrolytes. The activation energy (*E*_*a*_, eV) and pre-exponential factor (*σ*_*0*_, S cm^−1^) were fitted based on Eq. 2 (Arrhenius equation) [25, 26]:

2 $$\ln \sigma = \ln \sigma _0 - \frac{E_a}{{kT}}$$

where *σ* (S cm^−1^), *σ*_*0*_ (S cm^−1^), *E*_*a*_ (eV), *k*, and *T* (K) stand for the ionic conductivity, pre-exponential factor, activation energy, Boltzmann constant, and absolute temperature, respectively. The lithium migration number (*t*_*Li*+_) was calculated based on Eq. 3 [27, 28]:

3 $$tLi + = \frac{I_{ss}}{{I_0}} \times \frac{\Delta V - I_0 \times R_0}{{\Delta V - I_{ss} \times R_{ss}}}$$

where *I*_*0*_ (A) and *I*_*ss*_ (A) are the starting current and steady state current, respectively. *ΔV* (V) is polarization voltage. *R*_*0*_ (Ω) and *R*_*ss*_ (Ω) are the impedance before polarization and steady state impedance, respectively. See the Supporting Information for detailed measurement parameters.

## Results and Discussion

### Preparation of Composite Membranes and ASSPEs

**Fig. 1:**
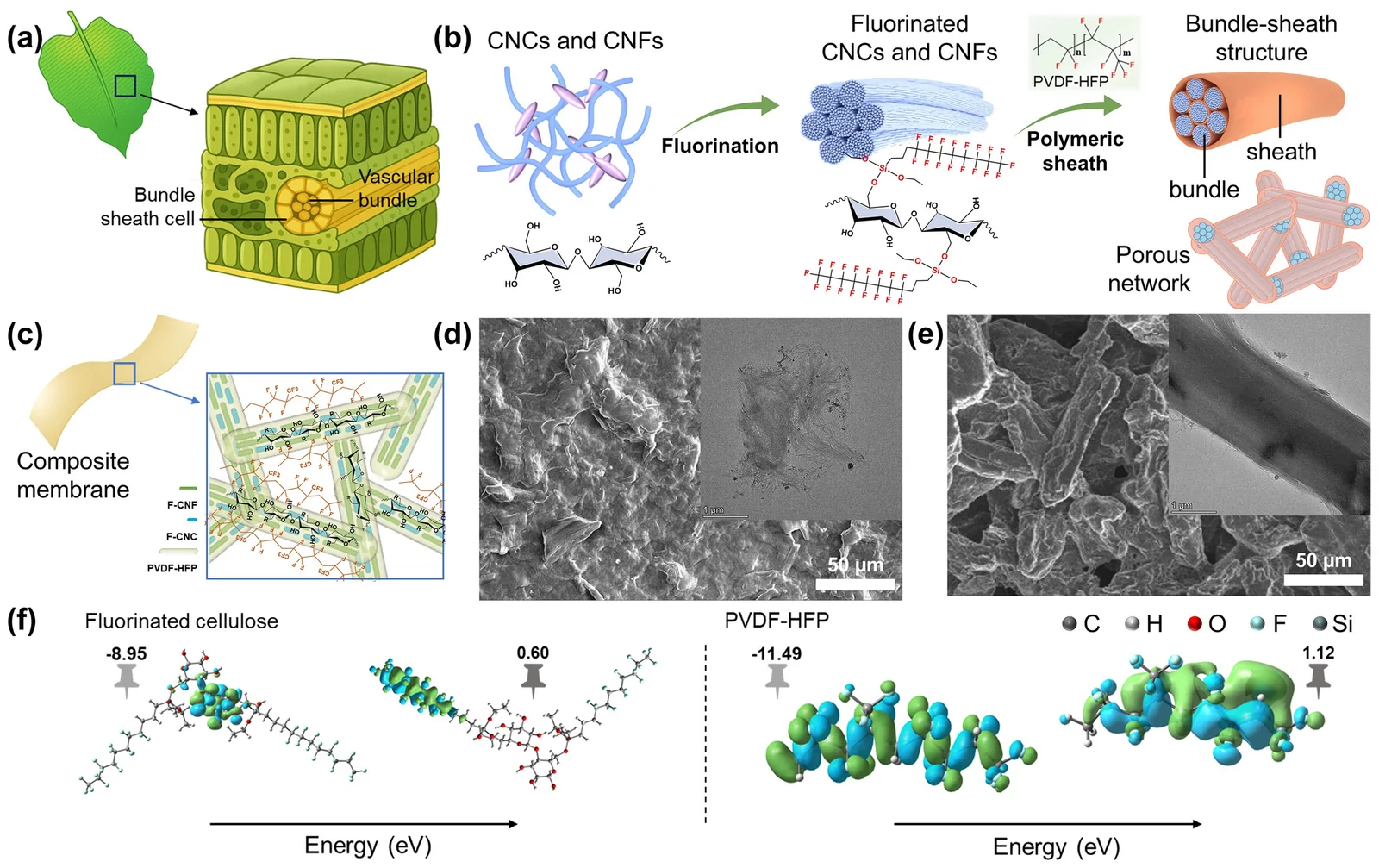
Schematic diagram of **a** vascular bundle, **b** formation of bundle-sheath structure, and **c** nanocellulose-based composite membrane. Surface SEM images of **d** membrane prepared from unfluorinated nanocellulose and PVDF-HFP and **e** FFP membrane (inset: TEM images of the microstructure). **f** HOMO and LUMO levels and orbitals

The vascular bundle is a plant structure that transports nutrients and is surrounded by vascular bundle-sheath cells for stability (Fig. 1a). Inspired by this naturally formed structure, a novel fluorinated nanocellulose/PVDF-HFP bundle-sheath structure and composite membrane were developed through shear-induced alignment (Fig. 1b, c). Specifically, fluorinated nanocellulose has a lower hydroxyl density, readily disperses in organic solvents, and unfolds its molecular chains [29]. As the solvent evaporates, uneven evaporation rates induce interfacial capillary pressure differences. Simultaneously, the localized viscosity increases, generating a local shear stress field, driving the F-CNFs with high aspect ratios to orient along the flow direction and pulling smaller F-CNCs [30–32]. The F-CNF and F-CNC molecular chains continuously connect into microfibrils, which further assemble into bundles. Moreover, the surface polarity and hydrophobicity of PVDF-HFP are similar to those of fluorinated nanocellulose, which prompts polymer chains to wrap bundles to form a sheath. The small-scale bundle-sheath structures assemble stepwise, forming the macroscopic units that constitute the composite membrane (Fig. S4). The stacked units form a porous network in the composite membrane to adsorb polymer precursors, while unfluorinated nanocelluloses aggregate in the polymer and form a dense structure (Figs. 1d, e and S5). This structural difference leads to a significant increase in the adsorption capacity of the composite membrane for polymer precursors (Fig. S6), which facilitates the formation of stable interfaces.

Under high-magnification TEM, an interlaced network composed of units and a highly regular, nearly parallel arrangement of fluorinated nanocelluloses were observed (Fig. S7). In contrast, unfluorinated nanocelluloses remain disordered, whether present alone or in mixtures (Fig. S8). This confirms that fluorination promoted nanocellulose orientation and the composite membrane was assembled from numerous ordered bundle-sheath structures. The abundant –CF_x_ groups in fluorinated nanocellulose can improve the electrochemical stability and promote the formation of stable SEI layers [33]. Furthermore, the pores between parallel nanocellulose bundles may serve as low-curvature channels for ion transport. These pores originate from the stacked nanocellulose molecules. Unlike the randomly distributed macropores in conventional porous membranes, this structure is more conducive to forming directional ion migration channels. During the in-situ polymerization step, the polymer electrolyte permeates and partially fills the pores. However, the parallel orientation remains intact, allowing ions to migrate rapidly along the fiber bundle direction.

The FT-IR spectrum of the composite membrane exhibits a split peak at 2889 cm^−1^, resulting from complex interactions (such as dipole–dipole interactions and van der Waals forces) between PVDF-HFP and fluorinated nanocellulose that alter the microenvironment of the C–H bonds. The peak at 1633 cm^−1^ shifted to the right, while the peak at 3460 cm^−1^ shifted significantly to the right and narrowed, indicating that the hydrogen bonds between nanocellulose molecules are partially replaced by interactions with PVDF-HFP (Fig. S9). This reflects a distinct interaction between the fluorinated nanocellulose and PVDF-HFP and the formation of the bundle-sheath structure. Figure 1f shows the HOMO and LUMO levels and orbitals of the fluorinated nanocellulose and PVDF-HFP. Both materials exhibit large energy band gaps, indicating that they have excellent electrochemical stability [34]. Furthermore, the lower LUMO level means that the SEI layers easily form on the anode surface, protecting the electrode and extending the cycle life [35]. The calculation results of the levels and orbitals further demonstrate the materials’ potential for use in battery separators.

The fabrication process of the composite membranes is inherently scalable. The parallel orientation of fluorinated nanocellulose forms naturally during solvent evaporation. This shear-induced alignment results in a robust bundle-sheath structure composed of fluorinated nanocellulose and PVDF-HFP. Crucially, the parallel orientation can be maintained from laboratory preparation to large-scale processing, requiring no special equipment or extreme processing conditions, meaning its feasibility for mass production. The shear-induced aligned bundle-sheath structures exhibit a stable morphology during solvent evaporation and maintain structural integrity after drying and under operational conditions. Fluorinated nanocellulose has enhanced chemical stability, while PVDF-HFP also demonstrates outstanding chemical resistance and stability. The bundle-sheath structure composed of fluorinated nanocellulose and PVDF-HFP offers excellent mechanical strength and thermal stability (Fig. S10). This enables the membranes to adapt to volume changes during cycling [36] and reduce the risk of thermal runaway. The crystallization behavior of PVDF-HFP is physically restricted by the nanocelluloses, and the decomposition of the composite membrane is a multi-step process. This feature also indicates that the composite structure has reliable long-term stability, and shear-induced alignment is not a transient effect but rather a persistent structural feature. This characteristic sufficiently ensures manufacturing feasibility and long-term stability under practical conditions.

The composite membranes served as separators for solvent-free in-situ polymerization, in which the polymer precursors were uniformly solidified to form ASSPEs (Fig. S11). VC dissolves other components and fully participates in curing as one of the monomers; octaMA-POSS serves as a crosslinking agent. VCIM-TFSI and TFA are used to promote lithium salt dissociation and enhance SEI layer stability. The FT-IR spectra revealed the disappearance of the C = C bond peak, evidencing complete polymerization (Fig. S12).

### Electrochemical Performance of ASSPEs

**Fig. 2:**
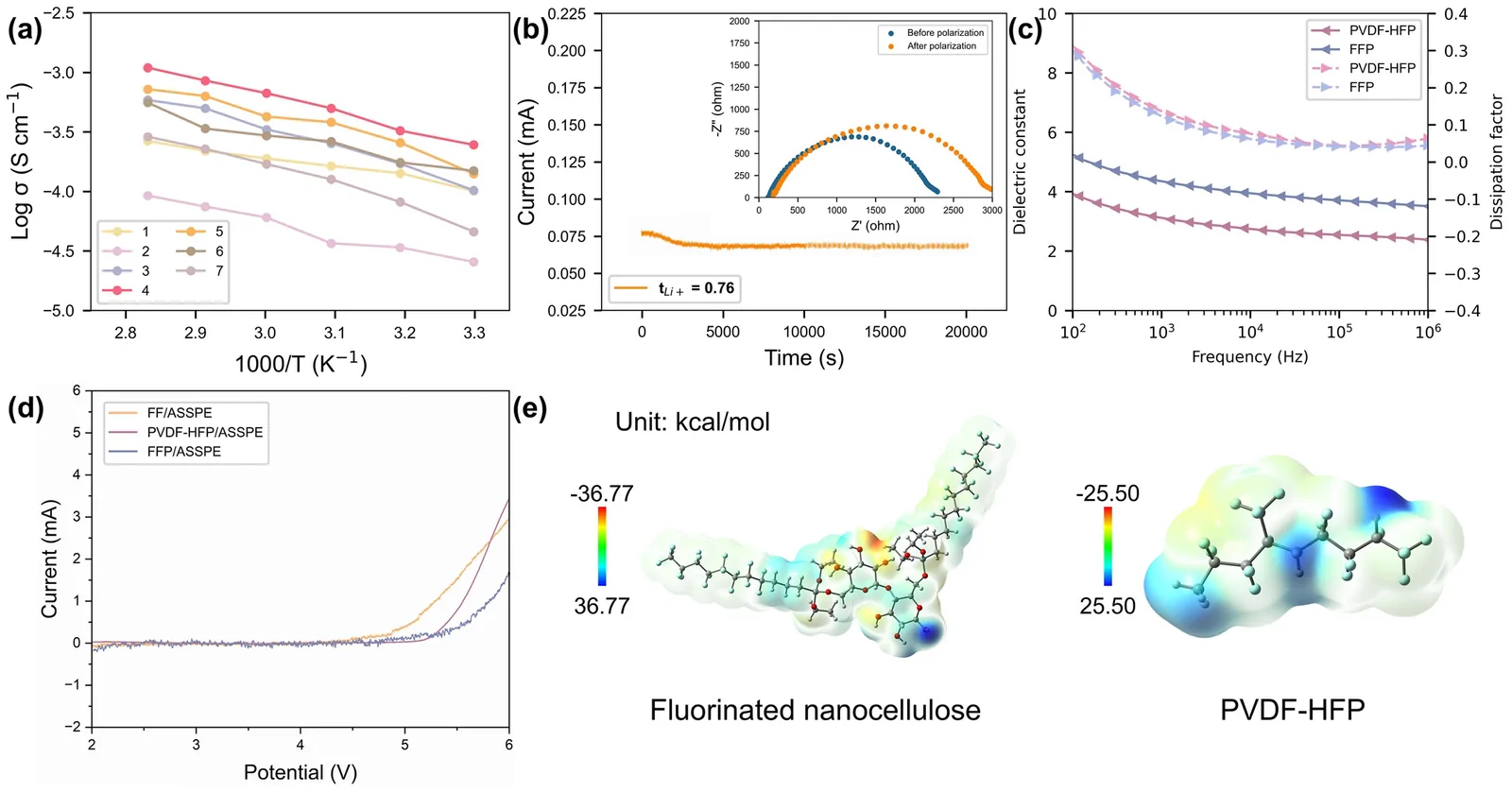
**a** Ionic conductivities and **b** lithium migration number of FFP/ASSPE (inset: the impedance before and after polarization). **c** Frequency dependence curves of the dielectric constant and dielectric loss of FFP. **d** LSV curves of FF/ASSPE, PVDF-HFP/ASSPE, and FFP/ASSPE. **e** Optimized geometric configurations and electrostatic potential of fluorinated nanocellulose and PVDF-HFP

The composition ratio (fluorinated nanocellulose and PVDF-HFP) of the composite membranes affects the bundle-sheath structure, which affects the ion transport and electrochemical stability of ASSPEs. The component ratios were optimized by orthogonal experiments. When the mass ratio of fluorinated nanocellulose to PVDF-HFP is 4:5 (number 4, Table S1), the constructed ASSPE (FFP/ASSPE) has the highest room temperature ionic conductivity of 2.46 × 10^−4^ S cm^−1^ (Fig. 2a). The data were fitted to the Arrhenius equation (Fig. S13 and Table S2). Ionic conductivity is considered to be influenced by the combined effects of *E*_*a*_ and *σ*_*0*_.* E*_*a*_ reflects the migration energy barrier for lithium ions, while the *σ*_*0*_ reflects the concentration of free lithium ions, the effective ion transport pathway, and the dielectric environment [37–39]. As the fluorinated nanocellulose content increases, the *E*_*a*_ and *σ*_*0*_ of the system first increase and then decrease. Upon the composite of fluorinated nanocellulose, lithium-ion migration transitions from chain-dependent motion to interfacial hopping transport, resulting in increased *E*_*a*_ [39–42]. The composite membranes with a high-dielectric constant promote lithium salt dissociation [43], increasing the concentration of free lithium ions. The highly oriented bundle-sheath structures form continuous ion transport channels. Besides, it is possible for a percolation effect to develop within the electrolyte when nanocelluloses interconnect and achieve sufficient structural integrity [44, 45]. These factors increased *σ*_*0*_. Group 4 is at the optimal equilibrium point, where the increase in *σ*_*0*_ outweighs the adverse effects caused by the rise in *E*_*a*_. Therefore, the ionic conductivity of Group 4 is optimal. As shown in Fig. 2b, the FFP/ASSPE also exhibits a high lithium migration number (0.76). A high migration number can reduce the concentration polarization, improve the fast charging capability, inhibit the growth of lithium dendrites, and extend the service life of batteries [46]. In comparison, the lithium migration numbers of FF/ASSPE and PVDF-HFP/ASSPE are 0.51 and 0.65, respectively (Fig. S14). The reasons for this difference are as follows: the PVDF-HFP sheath provides a high-dielectric (5.2, 100 Hz) environment that promotes the dissociation of lithium salts and increases the free lithium-ion concentration (Fig. 2c); parallel nanocellulose bundles form continuous low-curvature ion transport channels, enabling rapid lithium-ion migration along the bundle direction while suppressing the diffusion of larger anions within the confined space; and the PVDF-HFP in the composite membrane and positively charged imidazole rings in the polymer electrolyte can adsorb anions, inhibiting their movement. The synergistic effect of these factors inhibits the migration of anions, increasing the proportion of lithium ions involved in overall ion conduction. Moreover, fluorine-containing groups greatly improve the resistance to oxidative environments and inhibit interfacial side reactions, while the bundle-sheath structure improves the thermal stability and structural integrity of the system. These factors allow the electrolytes a wide electrochemical stability window (5.3 V, Fig. 2d), which could match with high-voltage electrodes. As shown in Fig. S15, the energy density of FFP increased, while its efficiency did not decrease significantly after the introduction of fluorinated nanocellulose. The superior high-voltage stability and electrochemical stability window enable greater capacity and energy density, promoting the miniaturization of all-solid-state lithium batteries. The electrostatic potential simulation results indicate that the active hydroxyl of fluorinated nanocellulose has a negative electrostatic potential, while the –CF_2_– groups of PVDF-HFP have a positive electrostatic potential (Fig. 2e). The PVDF-HFP sheath more readily captures TFSI^−^ anions, allowing more lithium ions to migrate freely within the membrane and electrolyte and increasing the ionic conductivity and lithium migration number. The analysis of electrostatic potential further demonstrates that the composite of fluorinated nanocellulose and PVDF-HFP can enhance the electrochemical performance of membranes and electrolytes.

**Fig. 3:**
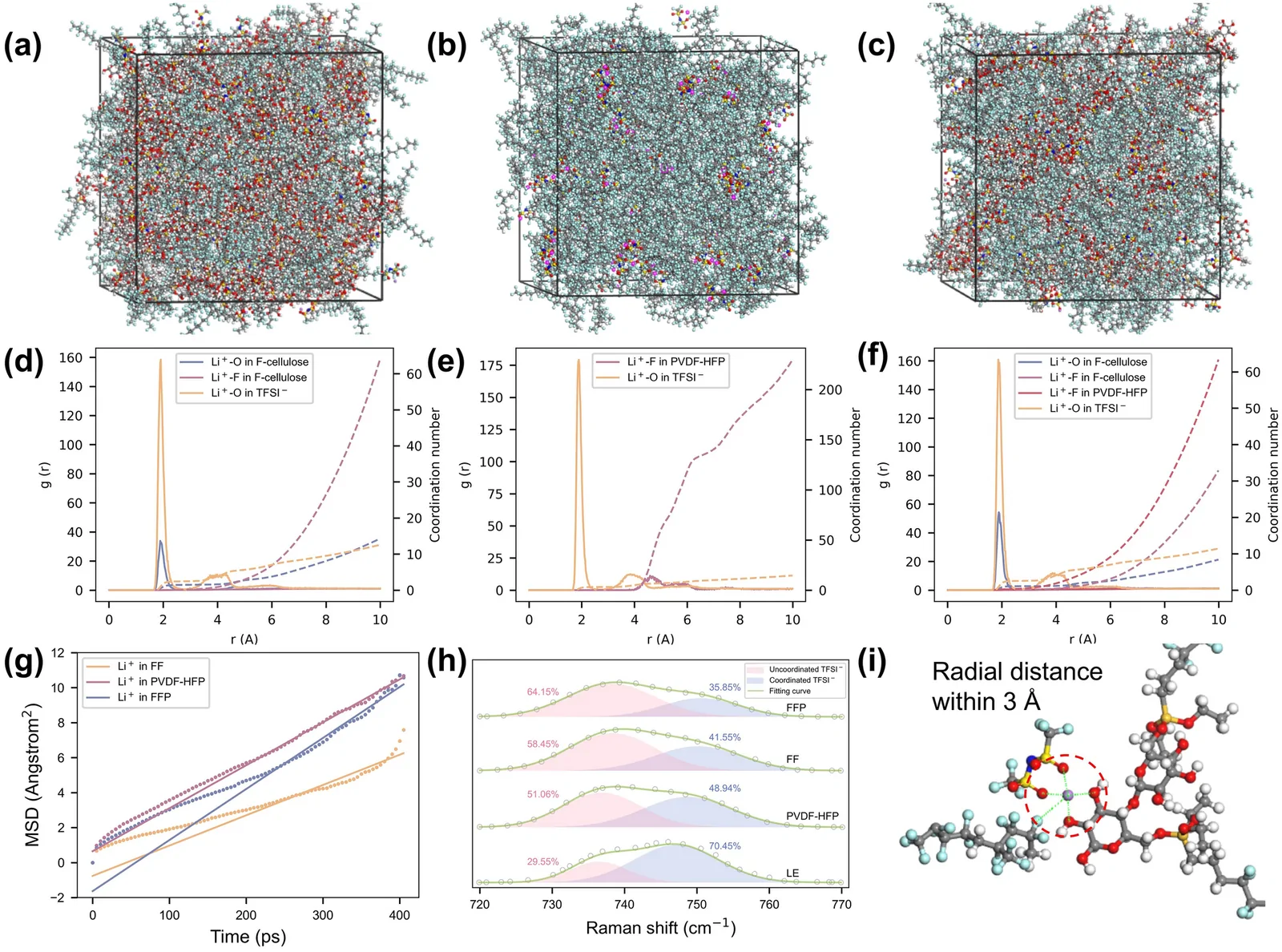
**a-c** Snapshots of MD simulation of FF, PVDF-HFP, and FFP. **d-f** Radial distribution function and coordination number of Li^+^–O/F in FF, PVDF-HFP, and FFP. **g** Ionic diffusion coefficients of FF, PVDF-HFP, and FFP. **h** Raman spectra of LE, FF, PVDF-HFP, and FFP. **i** O/F atoms within 3 Å of Li^+^ in optimized structural model

Molecular dynamics (MD) simulations were performed using software (Materials Studio 2023) to study the effect of the membrane microstructure on lithium-ion transport. The MD simulations of FF, PVDF-HFP, and FFP are shown in Fig. 3a–c. The radial distribution function (RDF) and mean square displacement (MSD) of the lithium ions in the membranes were simulated and calculated separately, using the lithium salt LiTFSI. The RDF was used to study the mutual distance and strength between lithium ions and groups in the membranes [47] (see Table S3 for detailed coordination numbers). The active oxygen atoms in nanocellulose and TFSI^−^ competitively coordinate with lithium ions, increasing the degree of dissociation of lithium salts (Fig. 3d). The Li^+^–O in TFSI^−^ coordination number in the PVDF-HFP membrane is only slightly higher than that in the nanocellulose membrane (Fig. 3e). The high density of fluorine atoms creates microenvironments with strong electronegativity and high dielectric, which can capture anions and increase the free lithium-ion concentration [48, 49]. Under the synergistic action of fluorinated nanocellulose and PVDF-HFP, the interaction between lithium ions and TFSI^−^ anions is weakened (Fig. 3f), and the lithium salts in the FFP membrane can effectively dissociate. This leads to a relatively high concentration of free lithium ions, which is beneficial for ionic conductivity and lithium migration number. The simulated Li^+^–F coordination number is also displayed, which stems from the statistical values resulting from the model configuration; lithium ions and fluorine atoms do not actually coordinate. The MSD reflects the diffusion behavior, phase transition characteristics, and motion patterns of particles [50]. On the basis of the MSD results, the diffusion coefficient was calculated to evaluate the ion transport capacity of the system and analyze the effect of structure on ion motion. The diffusion coefficient was fitted within the energy-stable range (200 ps ~ end). As shown in Fig. 3g, the lithium ions in the FFP membrane have the highest diffusion coefficient of 4.86 × 10^−7^ cm^2^ s^−1^ (in the FF membrane and PVDF-HFP membrane, they are 2.89 × 10^−7^ and 4.09 × 10^−7^ cm^2^ s^−1^, respectively). The reason for this difference is that the concentration of free lithium ions is highest in the composite membrane. Additionally, the bundle-sheath structure provides a fast ion transport path, which is conducive to lithium-ion migration. LiTFSI (in propylene carbonate and ethylene carbonate, 50/50, v/v) solution (1 M) was dropped onto different membranes, and the Raman spectra were obtained. The dissociation degree can be inferred from the fitted peak positions of TFSI^−^ anions uncoordinated (735 ~ 742 cm^−1^) and coordinated (747 ~ 753 cm^−1^) with lithium ions [51, 52]. The higher the proportion of uncoordinated TFSI^−^ anions, the greater the abundance of free lithium ions and the higher the degree of lithium salt dissociation. Raman spectra show that the uncoordinated TFSI^−^ anion in the FFP membrane is the highest (Fig. 3h), which is consistent with the above analysis (LE is the pure solution). The optimized structural model also demonstrates the competitive coordination between oxygen atoms in nanocellulose and TFSI^−^ with lithium ions (Fig. 3i). The results of molecular dynamics simulations indicate that composite membranes prepared from fluorinated nanocellulose and PVDF-HFP can promote lithium salt dissociation and improve ionic conductivity and lithium migration number.

### Cycling Performance of ASSLBs

**Fig. 4:**
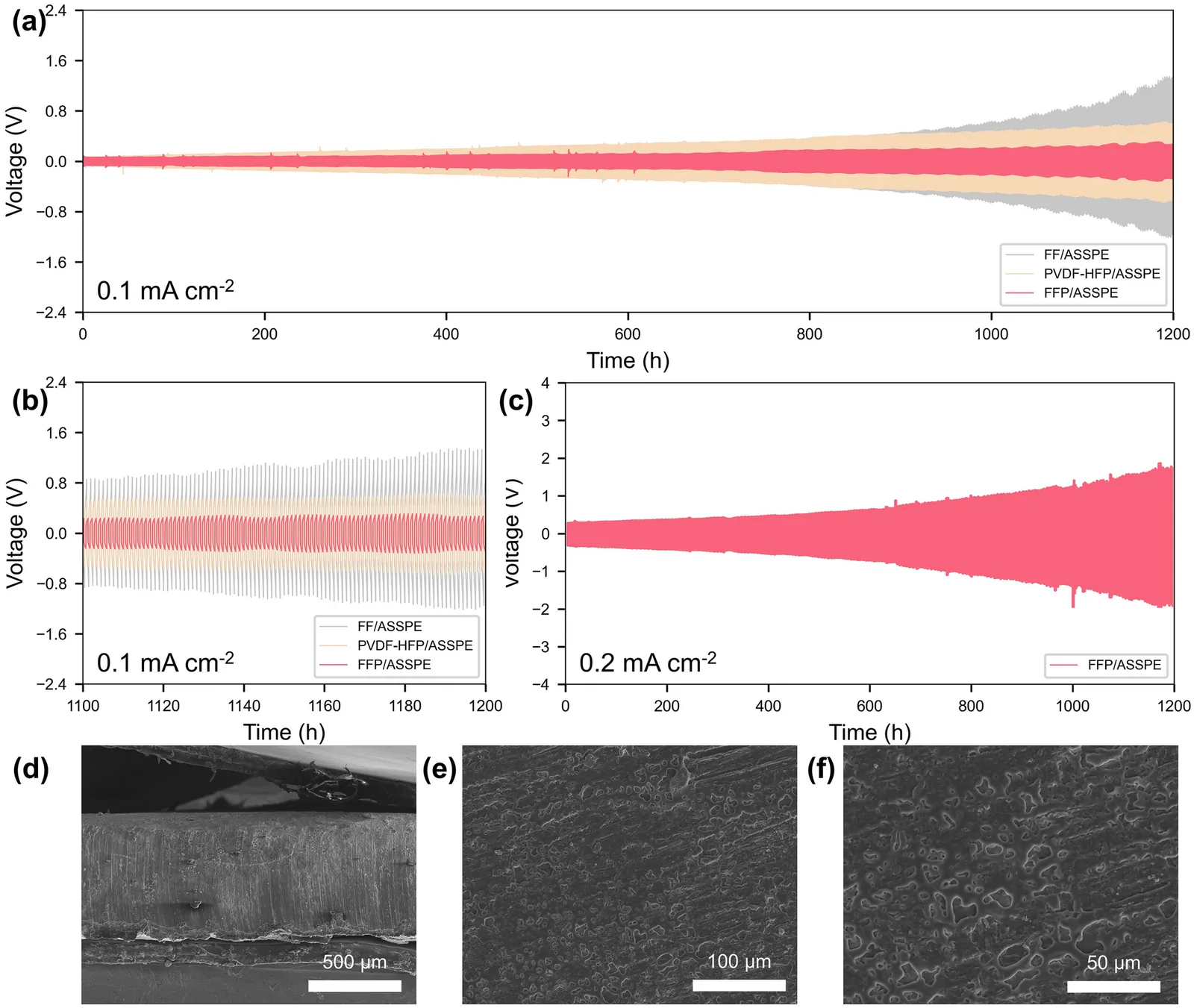
**a** Long-term cycling of Li||Li symmetrical cells at 55 °C. **b** Enlarged view of the 1100th-1200th cycle overpotential. **c** Long-term cycling of Li|FFP/ASSPE|Li cell at 55 °C. **d–f** SEM images of cross section and the surfaces of the lithium electrode of the Li|FFP/ASSPE|Li cell after 1200 h cycles at 0.1 mA cm^−2^

To study the effects of the composite membranes on the cell cycle stability, Li||Li symmetrical cells were assembled through in-situ polymerization and subjected to galvanostatic charge–discharge cycles. At a current density of 0.1 mA cm^−2^, the Li|FFP/ASSPE|Li cell has a significantly lower polarization voltage (Fig. 4a) and runs stably for more than 1000 h without significant polarization (Fig. 4b). The bundle-sheath structure achieves simultaneous optimization of ion transport channels and stable interfaces. Nanocellulose bundles serve as ion transport channels, promoting electrolyte adsorption and lithium-ion migration; the PVDF-HFP sheath provides mechanical and chemical stability and suppresses local concentration gradients. The synergistic effect of both components effectively reduces the polarization voltage of the symmetric cell, significantly slowing down lithium dendrite growth and the increase in interfacial impedance during cycling. The Li|FFP/ASSPE|Li cell can also operate at a high current density of 0.2 mA cm^−2^ for extended periods (Fig. 4c). Although the polarization voltage increases at the end of the cycle, a short circuit does not occur (Fig. S16). A possible reason is that the cell’s interfacial structure underwent detrimental changes as the cycle progressed, leading to an increase in impedance, hindering lithium-ion transport, and ultimately causing the polarization voltage to rise. As shown in Fig. 4d, the cross section of the lithium electrode of the Li|FFP/ASSPE|Li cell is smooth, without obvious dendrites after 1200 h cycles at 0.1 mA cm^−2^. The high-magnification SEM images also reveal uniform lithium deposition (Fig. 4e, f). In contrast, obvious lithium dendrites were observed in the SEM images of the lithium electrodes of the Li|FF/ASSPE|Li cell and Li|PVDF-HFP/ASSPE|Li cell (Fig. S17). The SEM results are consistent with the above analysis, further demonstrating that the bundle-sheath structures can improve the cycle stability and service life of cells.

**Fig. 5:**
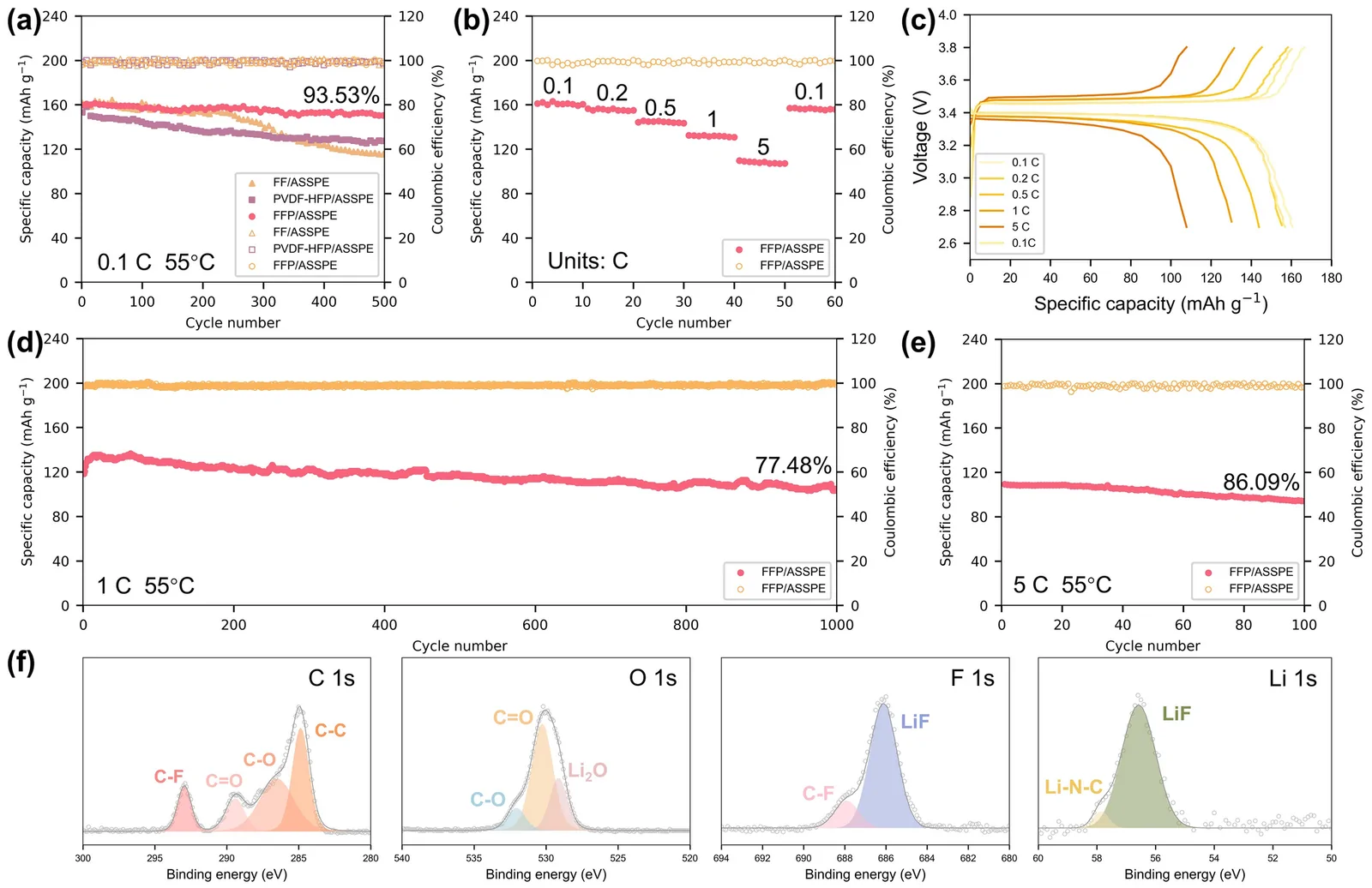
**a** Cycling and **b** rate performance of Li||LFP cells. **c** Charge–discharge curves of Li|FFP/ASSPE|LFP at different rates. **d** Long-time cycling performance of Li|FFP/ASSPE|LFP at 1 C. **e** Cycling performance of Li|FFP/ASSPE|LFP at a high rate of 5 C. **f** XPS spectra of lithium electrode after 1000 cycles of Li|FFP/ASSPE|LFP at 1 C

Figure 5 shows the cycling performance of the Li||LFP cells. After 500 cycles at 0.1 C and 55 °C, the capacity retentions of Li|FFP/ASSPE|LFP, Li|FF/ASSPE|LFP, and Li|PVDF-HFP/ASSPE|LFP are 93.53%, 71.47%, and 83.23%, respectively (Fig. 5a). The composite membranes can effectively suppress volume expansion during cycling and enable uniform stripping/electroplating of lithium ions, leading to improved cycle stability. Li|FFP/ASSPE|LFP also exhibits excellent rate performance and maintains stability at relatively high rates (Fig. 5b). As the rate increases, the Li|FFP/ASSPE|LFP capacity decreases steadily, showing good stability and reversibility (Fig. 5c). In addition, the capacity of Li|FFP/ASSPE|LFP can retain 77.48% after 1000 cycles at a high rate of 1 C, demonstrating practicality (Fig. 5d). The capacity of Li|FFP/ASSPE|LFP decreases steadily throughout the entire cycle process (Fig. S18), further demonstrating its excellent cycle stability. Notably, Li|FFP/ASSPE|LFP can even cycle 100 times at a high rate of 5 C and retain 86.09% of capacity (Fig. 5e). Compared with similar separators, the composite membrane FFP also offers advantages in terms of electrochemical performance (Table S4). Fluorinated nanocellulose and PVDF-HFP enable the electrolytes to adapt to a wide voltage window, enhancing the interface stability; the composite membranes provide high ionic conductivity and lithium migration number, slowing down the concentration polarization and local current unevenness inside the cells; the bundle-sheath structure enhances the mechanical strength and suppresses lithium dendrite growth and volume expansion. The combined effect of these factors ultimately led to a significant improvement in the cell cycle performance.

The XPS spectra of the lithium electrode after cycling are shown in Fig. 5f. The XPS spectrum of C 1*s* was fitted into four peaks: C–C (284.8 eV), C–O (286.5 eV), C = O (289.4 eV), and C–F (293.0 eV) [53]. The O 1*s* XPS spectrum has three peaks: Li_2_O (529.2 eV), C = O (530.3 eV), and C–O (532.1 eV) [54]. Li_2_O originates from the reaction between lithium and trace amounts of H_2_O/O_2_ in the electrolytes. A small amount of Li_2_O can promote the formation of an SEI layer and enhance the electrochemical stability. The peaks corresponding to LiF (686.2 and 56.6 eV) are also detected in the XPS spectra [55, 56], and the formation of LiF can effectively improve the cycle stability of cells. Additionally, PVDF-HFP and fluorinated nanocellulose participated in the interfacial reaction, resulting in C–F peaks in the spectra. The simultaneous presence of C–F and LiF indicates the formation of an inorganic–organic composite SEI layer, which has increased mechanical strength and electrical insulation, suppressing interfacial side reactions and dendrite growth [57, 58]. The Li–N–C originating from the imidazole ring in the polymer electrolyte is also observed in the Li 1*s* and N 1*s* spectra (Fig. S19), further indicating that the SEI layer is a multiphase structure composed of inorganic/organic components [59]. The XPS spectra indicate that fluorine-containing components in the electrolyte promote the formation of a multiphase SEI layer, which enhances cycle stability.

**Fig. 6:**
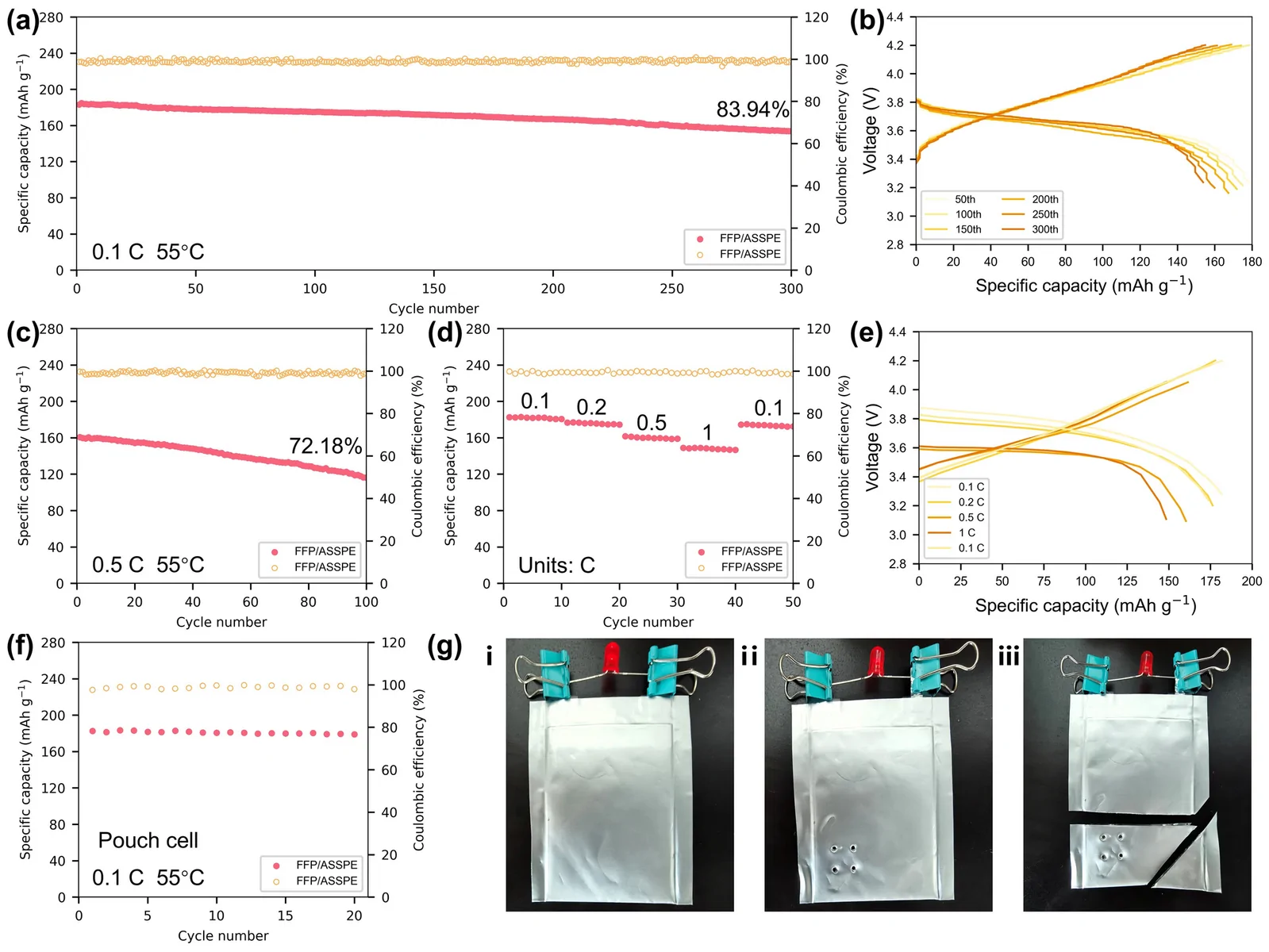
**a** Long-time cycling performance and **b** charge–discharge curves of Li|FFP/ASSPE|NCM811 at 0.1 C. **c** Cycling performance of Li|FFP/ASSPE|NCM811 at 0.5 C. **d** Rate performance of Li|FFP/ASSPE|NCM811. **e** Charge–discharge curves of Li|FFP/ASSPE|NCM811 at different rates. **f** Cycling performance of Li|FFP/ASSPE|NCM811 pouch cell. **g** Electronic photos of Li|FFP/ASSPE|NCM811 pouch cell lights up an LED during destructive testing

To further verify the application potential of the composite membranes in high-voltage systems, the cycle performance of Li||NCM811 cells was tested. The Li|FFP/ASSPE|NCM811 capacity decreases steadily and exhibits a capacity retention of 83.94% after 300 cycles at 0.1 C (Fig. 6a, b), indicating that the composite membranes can adapt to high-voltage conditions. Li|FFP/ASSPE|NCM811 also exhibits excellent rate performance and can run 100 times and retain 72.18% of capacity at 0.5 C, showing promise for use at higher rates (Fig. 6c–e). The fluorinated groups enhance the composite membrane’s antioxidant capacity and promote the formation of robust SEI layers [60, 61]. In addition, the bundle-sheath structure improves stability and prevents decomposition under high voltage. These factors allow the composite membranes to match with high-voltage cathodes and ensure structural stability during cycling. The cycle performance of Li||LCO cells at higher voltages can also serve as evidence (Fig. S20). Furthermore, Li|FFP/ASSPE|NCM811 pouch cells were prepared and subjected to short-term charge–discharge cycles (Fig. 6f). The pouch cells can cycle stably, indicating good interface compatibility between the electrolyte and electrodes and high electrochemical stability. As shown in Fig. 6g, the pouch cell can still light up an LED under extreme conditions, showing excellent stability and safety. The test results further indicate that the composite membranes can improve the interface stability, electrochemical stability, ion transport performance, and mechanical integrity and have application potential.

### Safe Performance of Pouch Cells

**Fig. 7:**
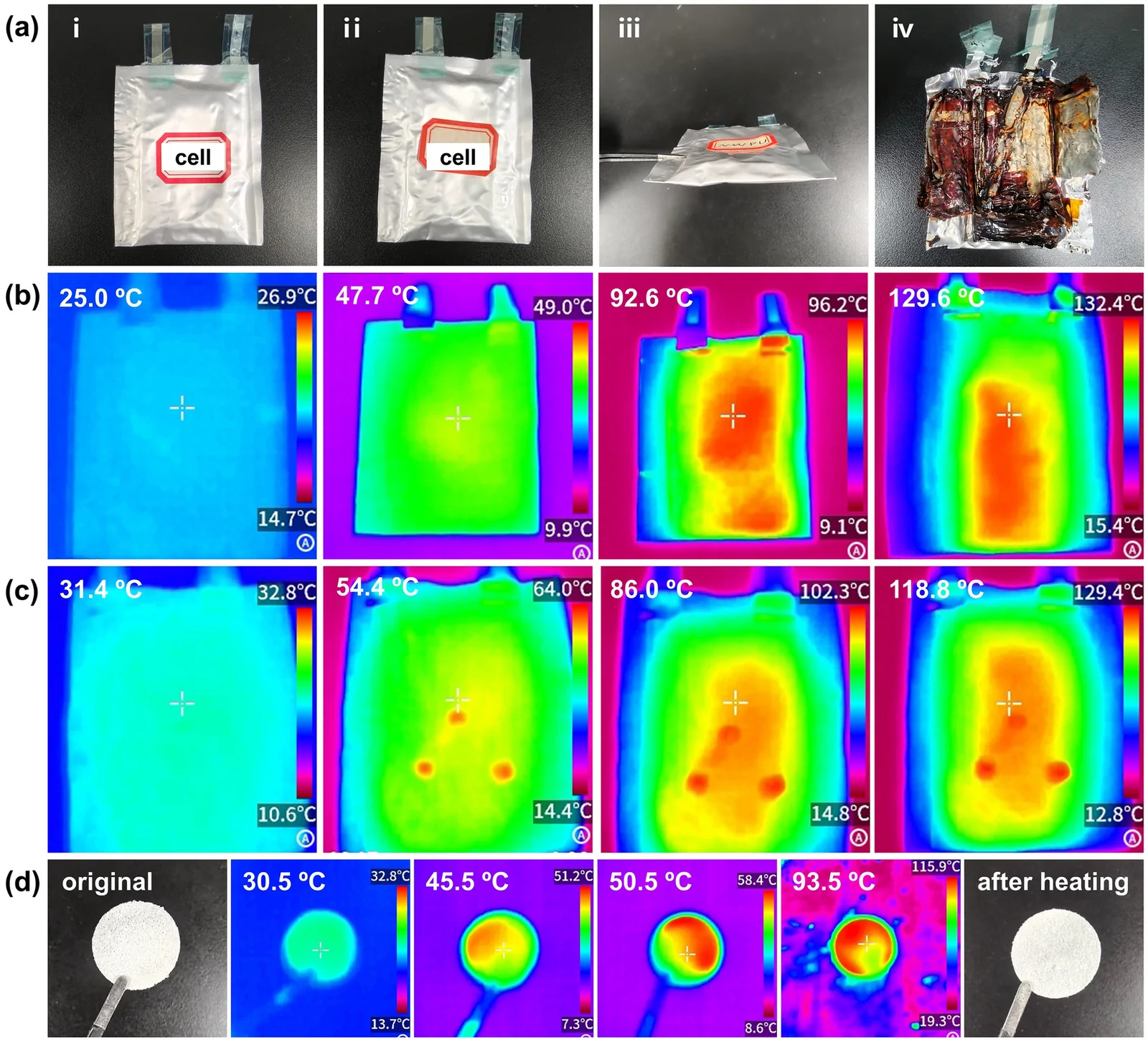
**a** Li|FFP/ASSPE|NCM811 pouch cell after thermal abuse test (original, post-test, side view, disassembled). Infrared thermogram of Li|FFP/ASSPE|NCM811 pouch cell **b** before and **c** after nail penetration. **d** Infrared thermogram of FFP at different temperatures (25 ~ 130 °C)

Figure 7 shows the safe performance of the Li|FFP/ASSPE|NCM811 pouch cell at high temperatures. After the thermal abuse test at 130 °C, the pouch cell showed no swelling, and the lithium electrode structure remained intact (Fig. 7a), indicating that the electrode/electrolyte interface is stable without significant side reactions. The cell surface temperature is uniformly distributed at low temperatures (30 and 50 °C). As the temperature increases above 100 °C, the heat is concentrated in the cell’s central region (Fig. 7b), which is attributed to uneven heat dissipation caused by the stacking structure and the preferential occurrence of interface reactions in the central area. To further validate the safety of the pouch cells, the thermal abuse test was performed on the punctured cell. There are distinct localized high-temperature areas around the cell’s puncture sites, but the overall temperature distribution pattern remains consistent with that observed without nail penetration (Fig. 7c). This result indicates that although nail penetration causes localized short circuits and heat generation, the cell system can confine these effects to the puncture sites, preventing the progression to a widespread thermal runaway. After heating to 120 °C, the composite membrane can maintain excellent dimensional stability and structural integrity (Fig. 7d), which is highly advantageous for thermal runaway protection in pouch cells, particularly in preventing interlayer short circuits. The electrolyte maintains structural integrity after heating, and the temperature on the electrolyte surface is always uniformly distributed (Figs. S21 and S22). The excellent thermal stability and safety result from the unique bundle-sheath structure of the composite membrane. The fluorinated nanocellulose bundles provide a high-decomposition-temperature backbone and continuous support network, effectively limiting the thermal softening and dimensional shrinkage of PVDF-HFP at elevated temperatures. Meanwhile, the PVDF-HFP sheath forms an interlocking structure around the bundles. This geometric constraint prevents structural failure when the polymer begins to soften. Furthermore, strong interfacial interactions between the fluorinated nanocellulose and PVDF-HFP prevent delamination under heat. As a result, the bundle-sheath structure enhances the dimensional and structural integrity of the membrane, leading to significantly improved thermal stability and safety of pouch cells.

## Conclusions

In summary, bioinspired fluorinated nanocellulose/PVDF-HFP bundle-sheath structure and composite membranes with high ionic conductivity and electrochemical stability have been prepared for long-cycle all-solid-state lithium batteries. The formation of the bundle-sheath structure originates from the intrinsic shear force field during the casting process, rather than from specialized equipment. It maintains a stable orientation structure and long-term stability even during scaled-up production. The low-curvature gaps between parallel nanocellulose molecule chains, microfibrils, and bundles promote the migration of lithium ions, while the PVDF-HFP sheaths improve stability and the dissociation of lithium salts. This unique bundle-sheath structure enhances the composite membrane’s mechanical strength (11.8 MPa) and endows the polymer electrolyte with a high room temperature ionic conductivity of 2.46 × 10^−4^ S cm^−1^. These properties enable the polymer electrolyte to suppress lithium dendrites and extend cycle life. The Li||Li cells can work for more than 1200 h at a current density of 0.2 mA cm^−2^ without short circuits. After 1000 and 100 cycles at high rates of 1 and 5 C, the Li||LFP batteries’ capacity could retain 77.48% and 86.09%. Furthermore, the bundle-sheath structure significantly enhances the system’s oxidation resistance, enabling the polymer electrolytes to withstand high voltages (5.3 V). The Li||NCM811 cells can maintain high capacity after prolonged high-voltage cycles (83.94% after 300 cycles at 0.1 C, 72.18% after 100 cycles at 0.5 C). This strategy holds promise for enabling the application of cellulose separators in high-performance all-solid-state lithium batteries.

## Supplementary Information

Below is the link to the electronic supplementary material.

Supplementary file1 (DOCX 16589 kb)
