# Supplementary material for: Bioinspired Vascular Bundle Structured Nanocellulose/PVDF-HFP Composite Membranes for Efficient Ion Transport and Stable All-Solid-State Lithium Batteries
Source: Nanomicro Lett. 2026 Feb 14;18:254. doi: 10.1007/s40820-026-02092-0 (PMC12906618; doi:10.1007/s40820-026-02092-0)
Supplement: Supplementary file 1 — Supplementary file1 (DOCX 16589 kb) [file 40820_2026_2092_MOESM1_ESM.docx]

Supporting Information for

**Bioinspired Vascular Bundle Structured Nanocellulose/PVDF-HFP Composite Membranes for Efficient Ion Transport and Stable All-Solid-State Lithium Batteries**

Chenxiang Gao^1^, Yijie Zhou^1^, Yun Huang^2^, Shuhui Wang^1^ and Xiaoyan Ma^1,^ *

^1^ School of Chemistry and Chemical Engineering, Northwestern Polytechnical University, Xi'an 710072, P. R. China

^2^ School of New Energy and Materials, Southwest Petroleum University, Chengdu 610500, P. R. China

*Corresponding author. E-mail: [m_xiao_yana@nwpu.edu.cn](mailto:m_xiao_yana@nwpu.edu.cn) (Xiaoyan Ma)

**S1 Experimental Section**

**S1.1 Materials and Chemicals**

Alpha-cellulose (particle size = 25 µm, Rharn), concentrated sulfuric acid (sinopharm), 2,2,6,6-tetramethylpiperidinooxy (TEMPO, ≥98%, aladdin), sodium bromide (NaBr, 99.9%, Rharn), sodium hypochlorite (NaClO, active chlorine 10%, Rharn) and ethanol anhydrous (AR) were used to prepare fluorinated nanocelluloses. Sodium hydroxide (NaOH, AR, Rharn) and dilute hydrochloric acid were used to regulate pH. 3-(methacryloxy)propyltrimethoxysilane (AR, Macklin), tetramethylammonium hydroxide (TMAH, Rharn), isopropanol (AR, 99.5%, Rharn), methylbenzene, saturated saline and magnesium sulfate anhydrous (AR, Rharn) were used for the preparation of octamethacryloyloxypropyl polyhedral oligomeric silsesquioxane. 1-vinylimidazole (≥99%, aladdin), 5-chloropentanenitrile (98%, Rharn), lithium bis((trifluoromethyl)sulfonyl)azanide (LiTFSI, 99%, OriLeaf) and ethyl acetate (sinopharm) were used for the preparation of 1-vinyl-3-pentyl cyano imidazole (trifluoromethanesulfonyl) imide. Ferrous lithium phosphate (LiFePO_4_, LFP, ≥99%, aladdin), NCM811 (Canrd), LCO (LiCoO_2_, Canrd), poly(vinylidene fluoride) (PVDF), acetylene carbon black (SUPER P, TIMCAL) and N-methyl-2-pyrrolidone (NMP, 98%, Rharn) were used to prepare electrodes.

**S1.2 Preparation of cellulose nanocrystals**

Cellulose nanocrystals (CNCs) were prepared by dispersing alpha-cellulose (5 g) in sulfuric acid (64 *wt*%, 100 mL) and acidifying for 30 min at 45 ℃ [S1]. Wash CNCs with deionized water until neutral, then freeze-dry and grind them, and store at low temperatures.

**S1.3 Preparation of cellulose nanofibers**

Cellulose nanofibers (CNFs) were prepared by the oxidation method. Briefly, alpha-cellulose (2 g) was dispersed in the solution (0.032 g TEMPO and 0.2 g NaBr) and stirred for 2 h. Oxidation reactions were triggered by adding NaClO (5 mL) and adjusting the pH (maintained at about 10). When the pH no longer changes significantly, the reaction is complete [S2]. Wash CNFs with deionized water until neutral, then freeze-dry and grind them, and store at low temperatures.

**S1.4 Preparation of fluorinated nanocellulose membranes**

F-CNC and F-CNF (mass ratio 1:3) were dispersed in deionized water and stirred for 2 h at 60℃. Then, the dispersion was vacuum filtered to form membranes. Finally, freeze-dry the membranes for 24 h and dry them at 80℃ for 24 h to obtain the fluorinated nanocellulose membranes (recorded as FF).

**S1.5 Synthesis of octamethacryloyloxypropyl polyhedral oligomeric silsesquioxane**

Firstly, the isopropanol solution of 3-(methacryloxy)propyltrimethoxysilane was dropped into the isopropanol solution of tetramethylammonium hydroxide at room temperature and stirred for 2 h. Then, the mixed solution was rotary evaporated to obtain white oily liquids. These liquids were dissolved in methylbenzene, washed to neutrality with saturated saline, dewatered by magnesium sulfate anhydrous, filtered, and rotary evaporated to obtain the purified product. This purified product and tetramethylammonium hydroxide were dissolved in methylbenzene and reacted at 120℃ for 4 h. Then, the reaction solution was cooled to room temperature and washed to neutrality with saturated saline. Finally, colorless viscous octaMA-POSS was obtained after anhydrous magnesium sulfate dewatering, filtration, rotary evaporation, and vacuum drying at 60 ℃ for 12 h [S3].

**S1.6 Synthesis of 1-vinyl-3-pentyl cyano imidazole (trifluoromethanesulfonyl) imide**

Briefly, 1-vinylimidazole and 5-chloropentanenitrile were mixed well and reacted at 70℃ for 12 h to obtain the crude product. This product was washed several times with ethyl acetate and dissolved in deionized water. An excess of LiTFSI was added to the solution and stirred for 4 h. The solution was then washed with a large of deionized water, and the lower liquid layer was separated. Finally, the lower liquid was dried under vacuum at 80℃ for 24 h to obtain a light yellow viscous liquid [S4].

**S1.7 Preparation of electrodes**

First, the LiFePO_4_ (0.1 g, or NCM811, LCO) was dispersed in NMP (3.5 g) and stirred overnight. Then, the dried SUPER P was added and stirred for more than 4 h. Finally, the dried PVDF was added and continued to be stirred for more than 4 h to obtain the electrode slurry. The slurry is scraped onto the aluminum foils and dried sufficiently to obtain the electrodes. Each electrode (without aluminum foil) was about 1.0~1.4 mg.

**S1.8 Measurements**

The solution used for contact angle testing is polymer precursor, and each drop is approximately 2 µL.

FT-IR was tested at wavenumbers from 400~4000 cm^-1^.

Ramam spectra were tested in the range of 730~760 cm^-1^.

TGA was performed under the following conditions: nitrogen atmosphere, heating rate of 10 ℃ min^-1^, temperature rise range of 25~800℃.

DSC was performed under nitrogen atmosphere with heating rates of 10 ℃ min^-1^.

The mechanical properties of the materials (the size of test samples is 8 mm × 2 mm) were evaluated at a tensile rate of 0.05 mm s^-1^ using a homemade tensile testing machine.

The testing frequency range of broadband dielectric spectrometer is 10^2^~10^6^ Hz.

The D-E loops of membranes were measured at an oil bath (room temperature) and the boost rate was 50 V s^-1^.

The bulk resistances of the electrolytes at 30~80℃ and in the frequency range of 1~10^6^ Hz were tested to calculate the ionic conductivities. The two electrodes of the cells are steel/steel.

The voltage range and scan rate of LSV were 2~6 V and 10 mV s^-1^, respectively. The two electrodes of the cells are steel/Li.

The lithium migration number was calculated from current polarization at constant voltage (10 mV) and cell impedance data before and after polarization (frequency range is 1~10^6^ Hz). The two electrodes of the cells are Li/Li.

The symmetrical cells operated under 0.1~0.2 mA cm^-2^ and 55℃. The two electrodes of the cells are Li/Li.

The full cells operated at different rates (0.1, 0.2, 0.5, 1, 5 C) and 55℃ in the voltage range of 2.7~3.8 V, 3.0~4.2 V and 3.0~4.4 V. The electrodes of the cells are LFP, NCM811, or LCO/Li.

Thermal abuse test is put the fully charged pouch cell into a test chamber, increase the temperature at a rate of 5 ± 2 ℃ until 130℃, and keep it for 30 min.

**S2 Supplementary Figures and Tables**
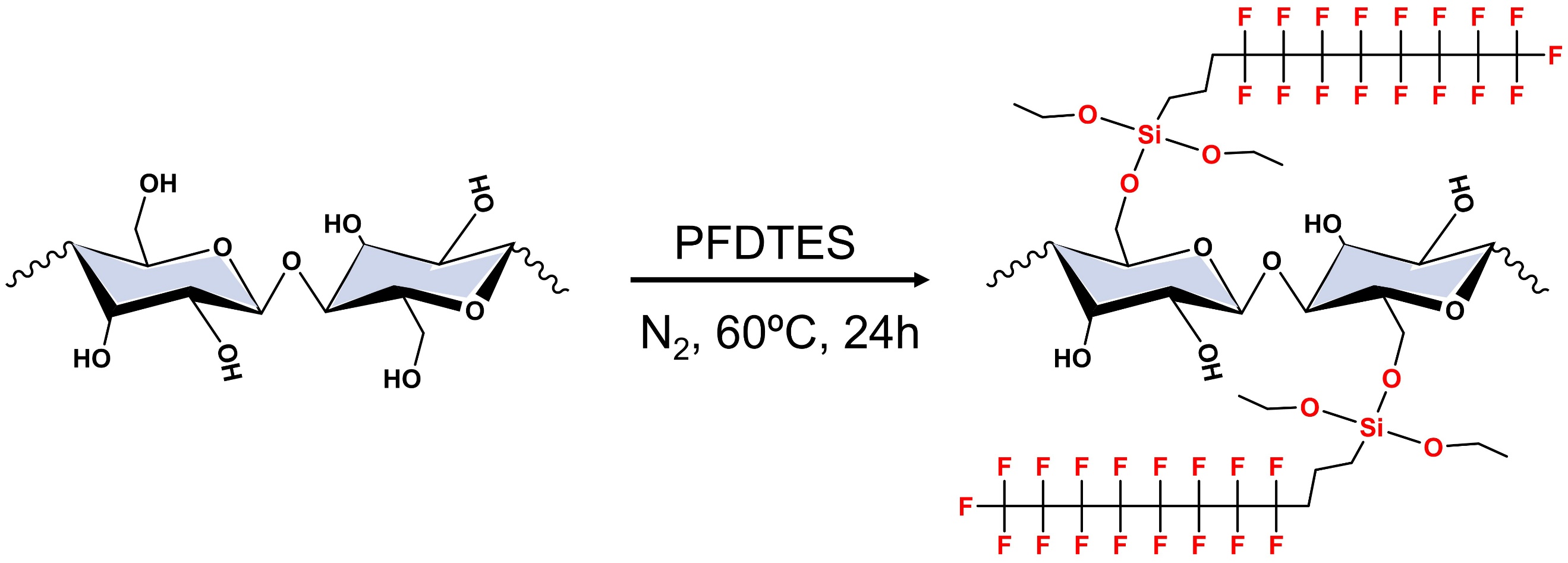


**Fig. S1** Preparation of fluorinated nanocelluloses


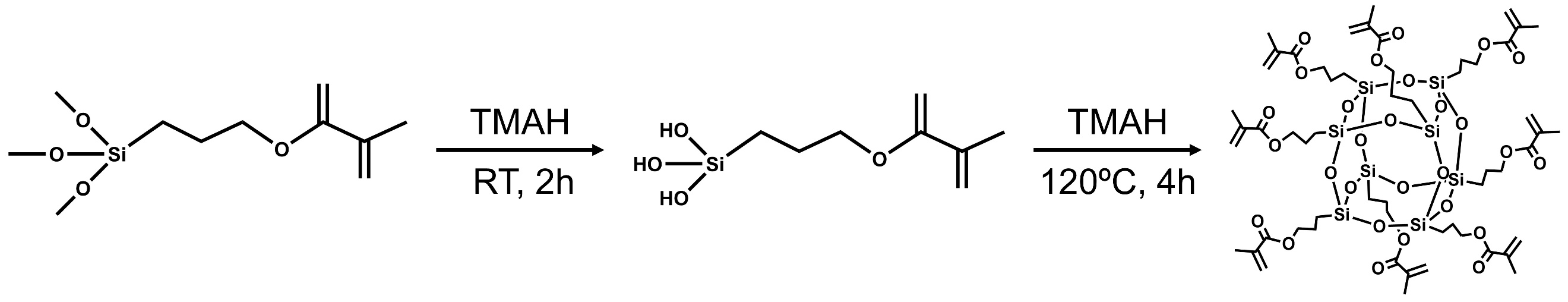


**Fig. S2** Synthesis of octamethacryloyloxypropyl polyhedral oligomeric silsesquioxane


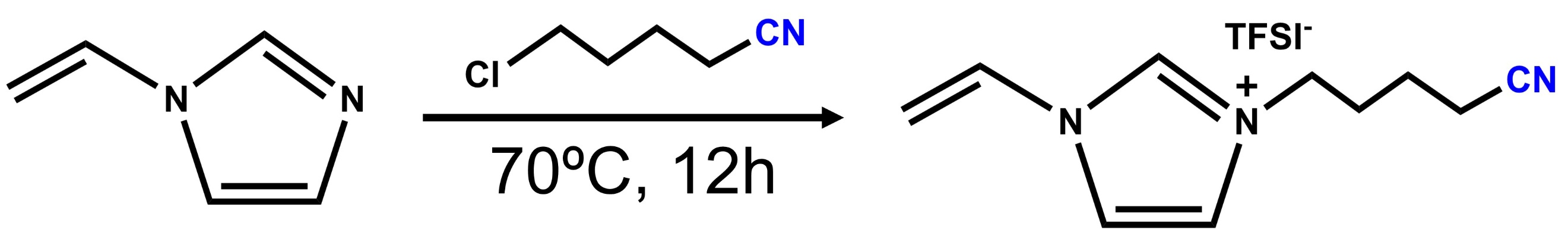


**Fig. S3** Synthesis of 1-vinyl-3-pentyl cyano imidazole (trifluoromethanesulfonyl) imide


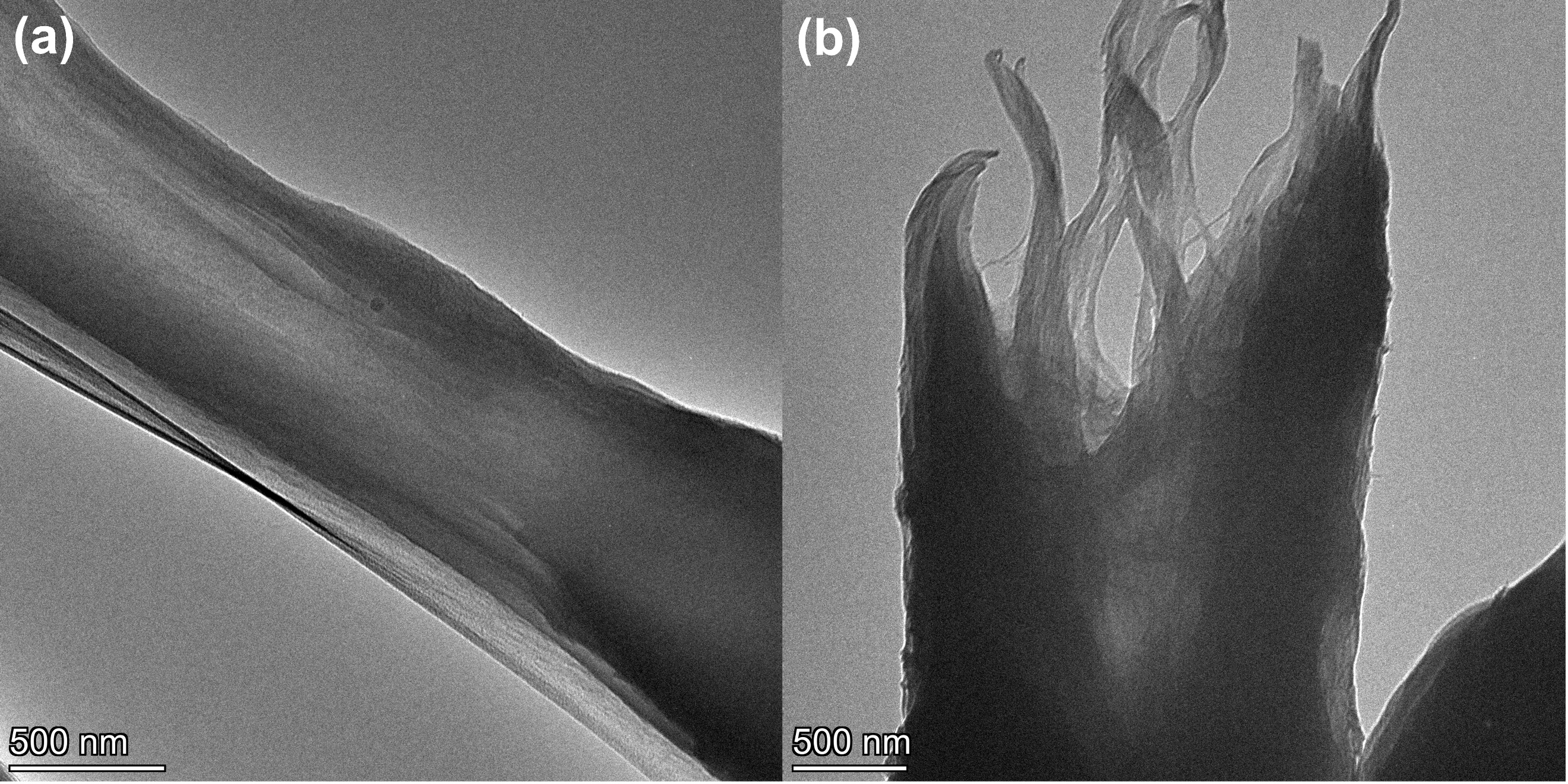


**Fig. S4** TEM images of (**a**) assembled bundle-sheath structure and (**b**) fracture surface


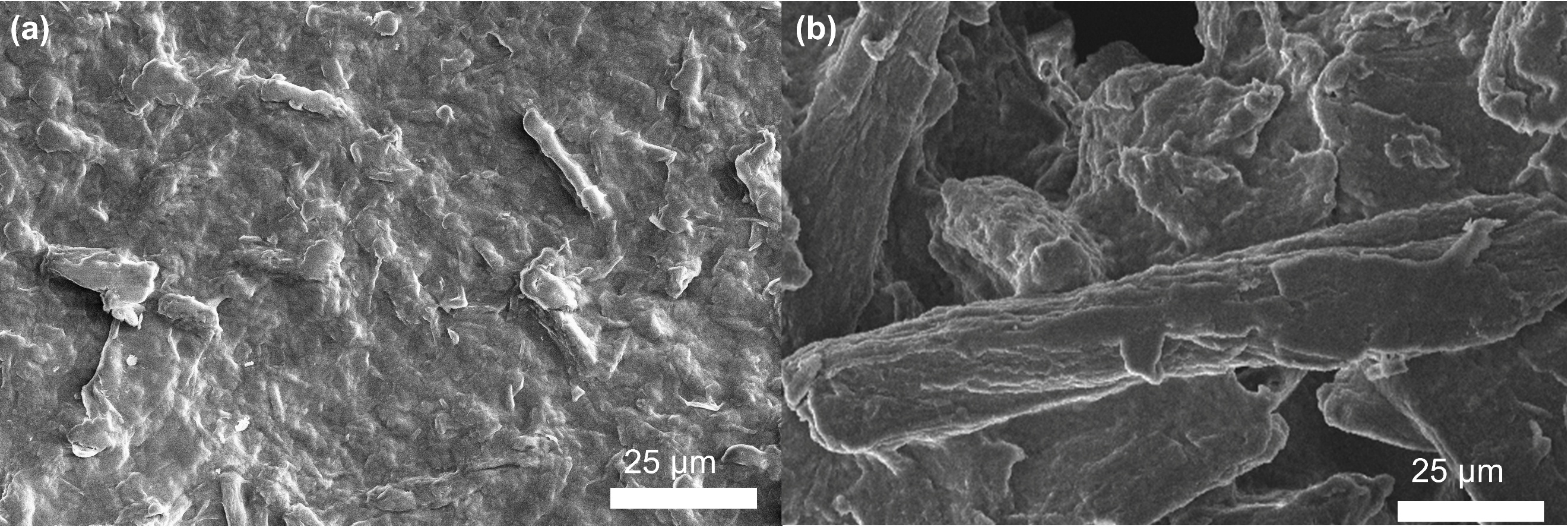


**Fig. S5** The high-magnification surface SEM images of (**a**) membrane prepared from unfluorinated nanocellulose and PVDF-HFP and (**b**) FFP membrane


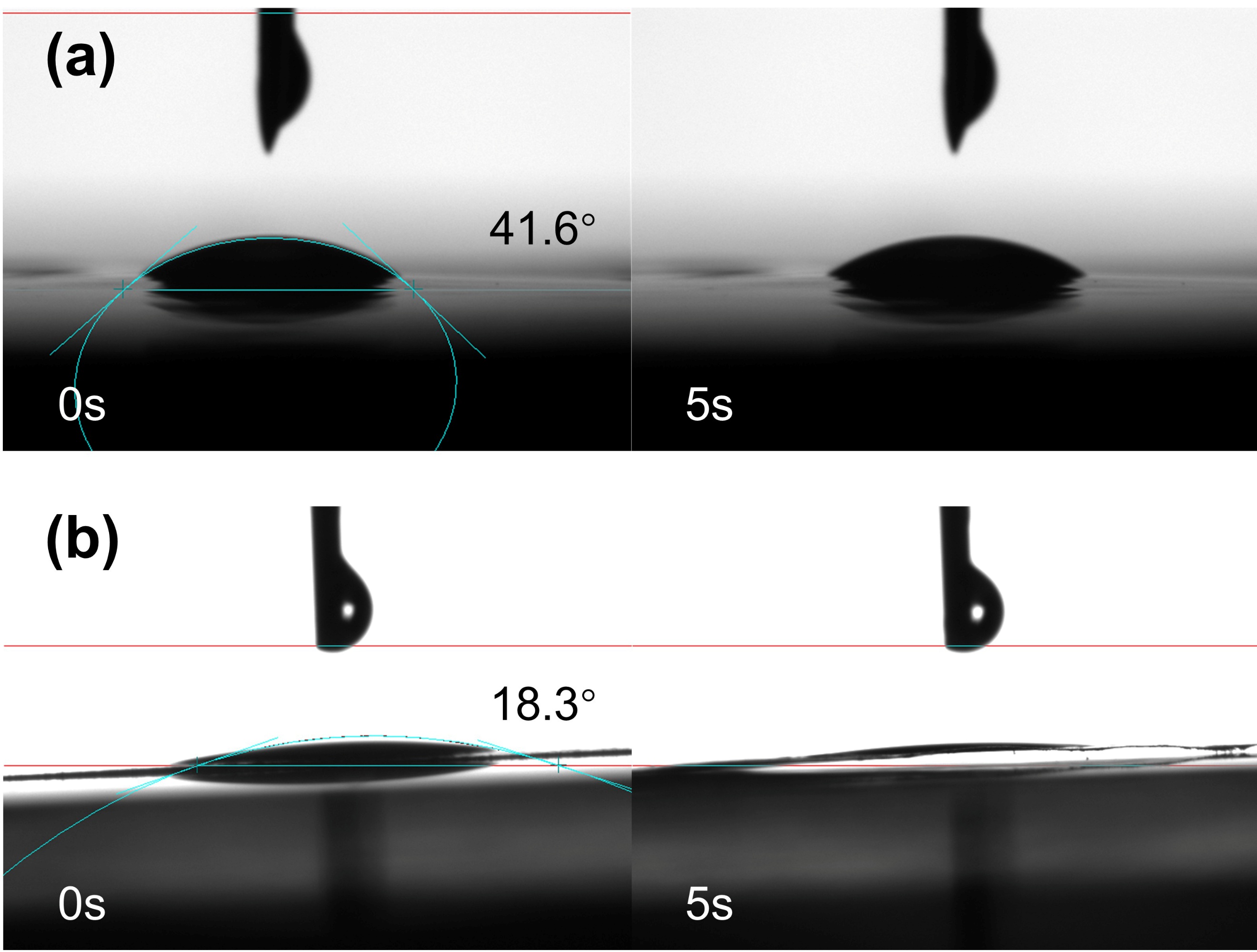


**Fig. S6** Contact angles of (**a**) membrane prepared from unfluorinated nanocellulose and PVDF-HFP and (**b**) FFP membrane


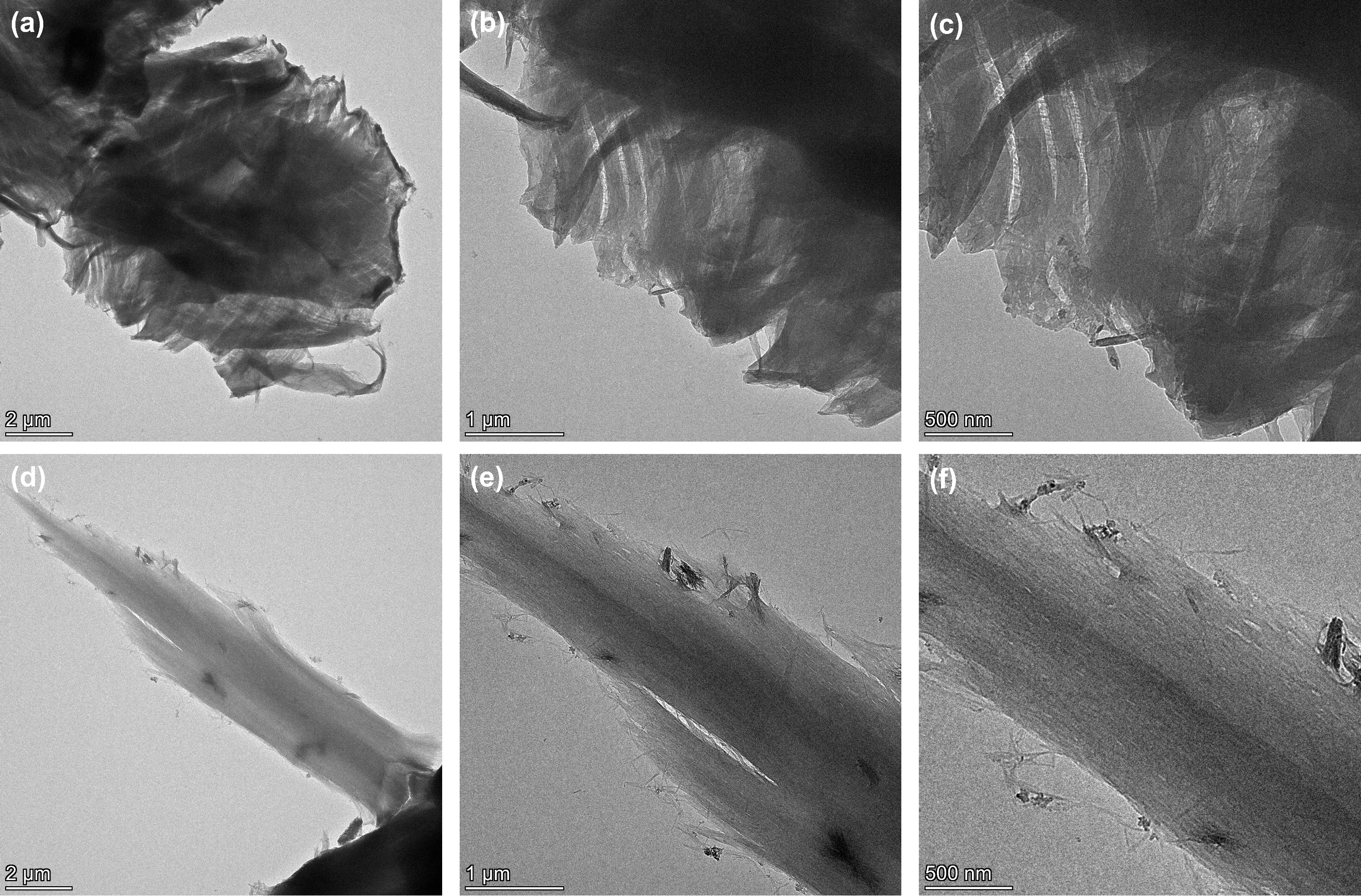


**Fig. S7** TEM images of (**a-c**) FFP membrane and (**d-f**) fluorinated nanocellulose


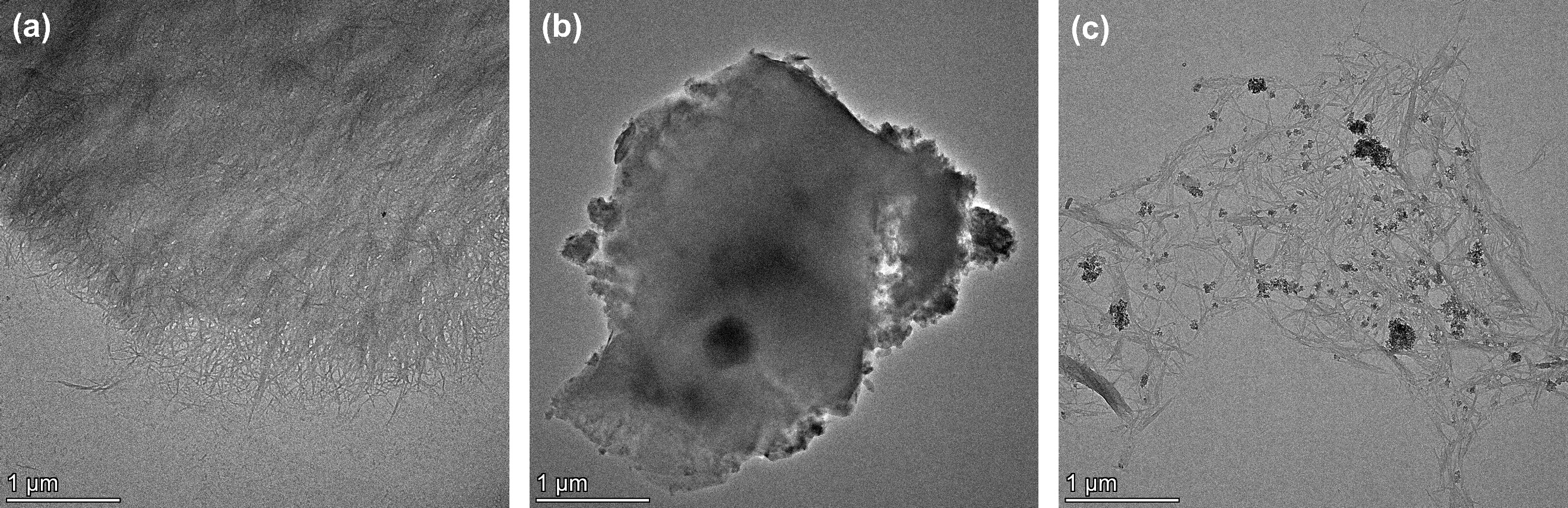


**Fig. S8** TEM images of (**a**) CNFs, (**b**) CNCs, and (**c**) a mixture of CNF/CNC


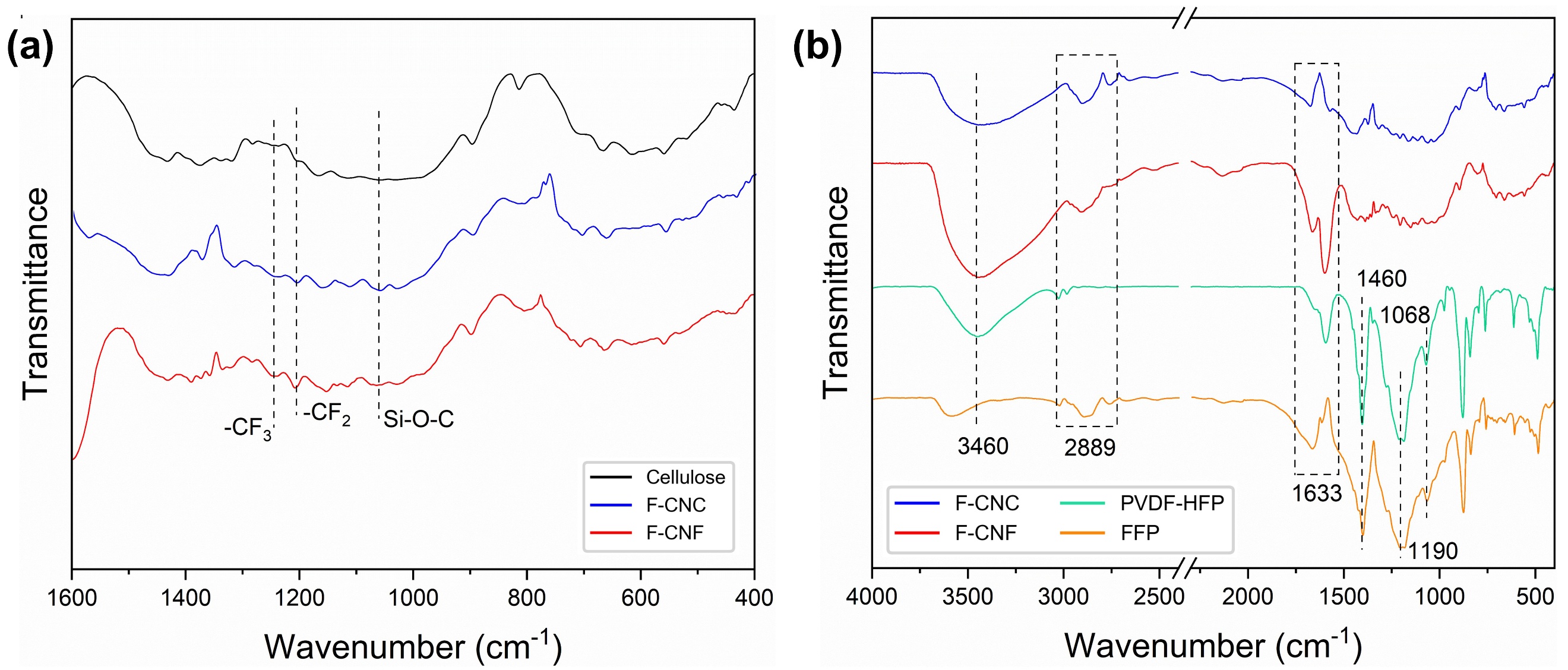


**Fig. S9** FT-IR spectra of (**a**) fluorinated nanocelluloses and (**b**) FFP membrane


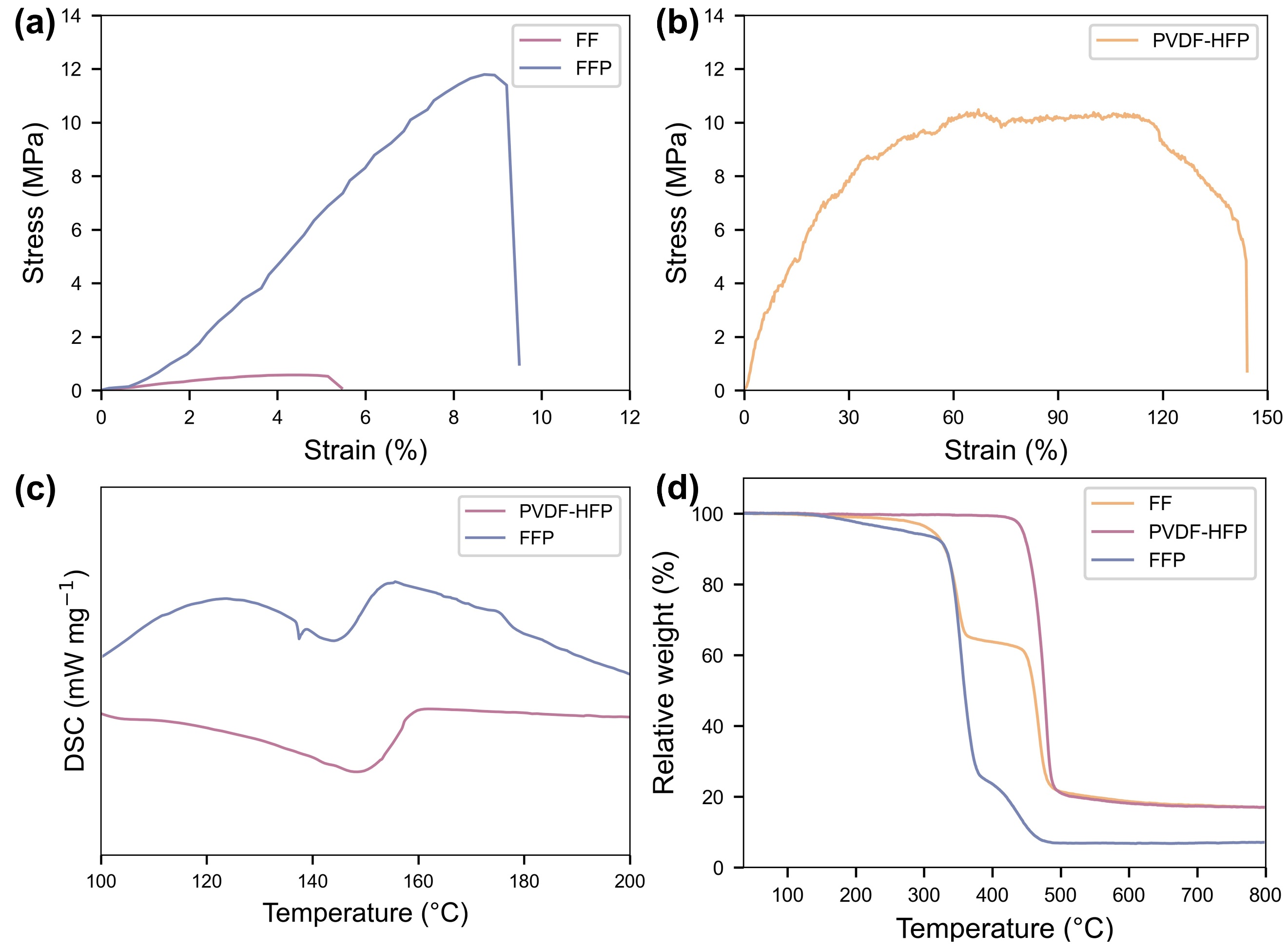


**Fig. S10** Stress-strain curves of (**a**) FF, FFP and (**b**) PVDF-HFP membrane. (**c**) DSC curves of PVDF-HFP and FFP. (**d**) TGA curves of FF, PVDF-HFP and FFP


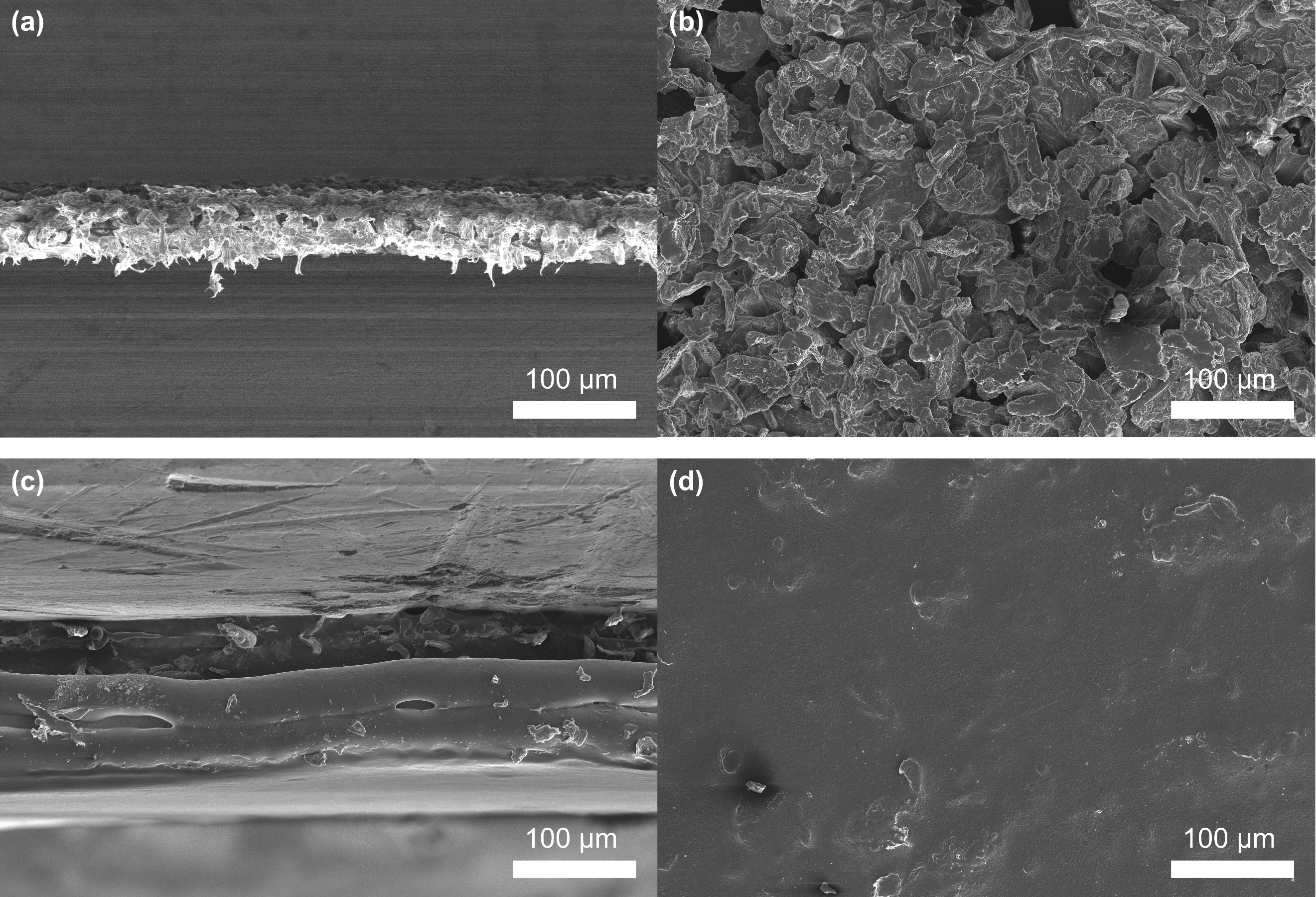


**Fig. S11** (**a**) Cross-section and (**b**) surface SEM images of FFP membrane. (**c**) Cross-section and (**d**) surface SEM images of FFP/ASSPE

**
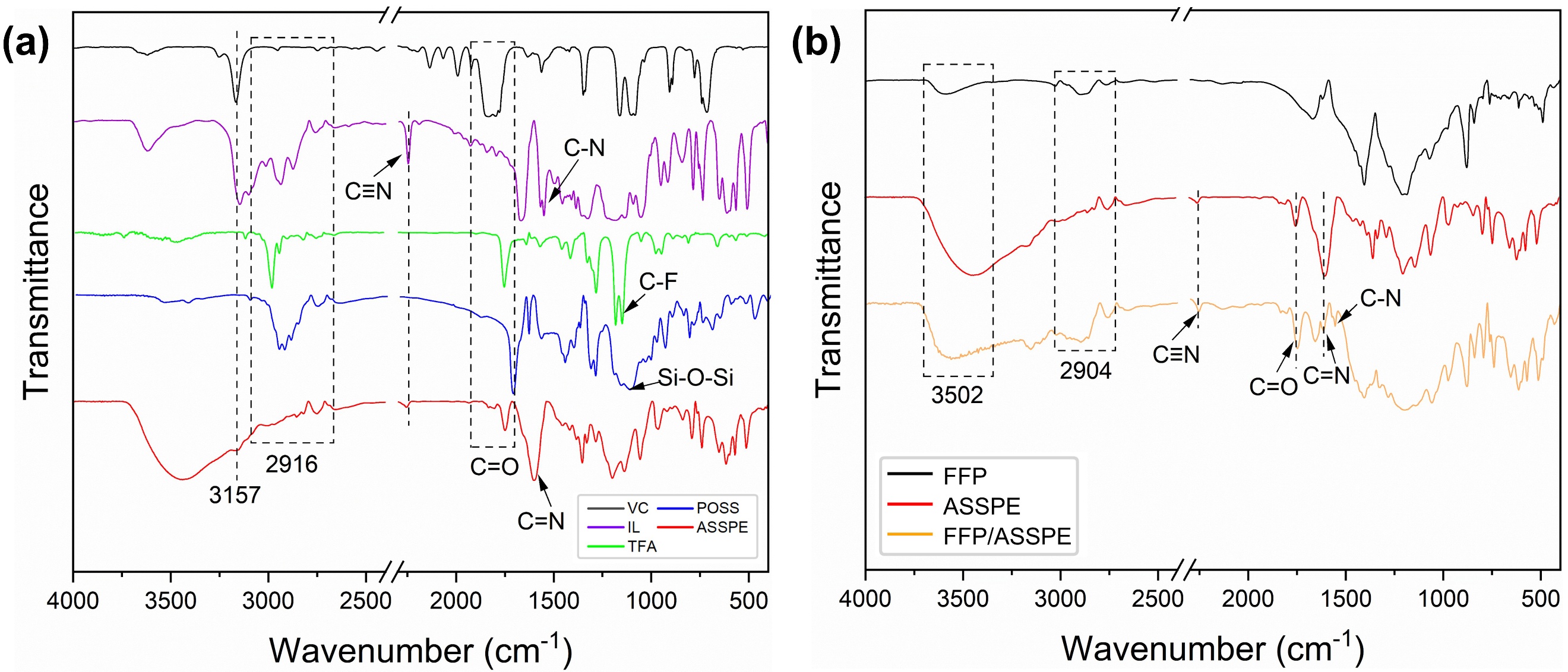
**

**Fig. S12** FT-IR spectra of (**a**) monomers and (**b**) FFP, ASSPE, FFP/ASSPE


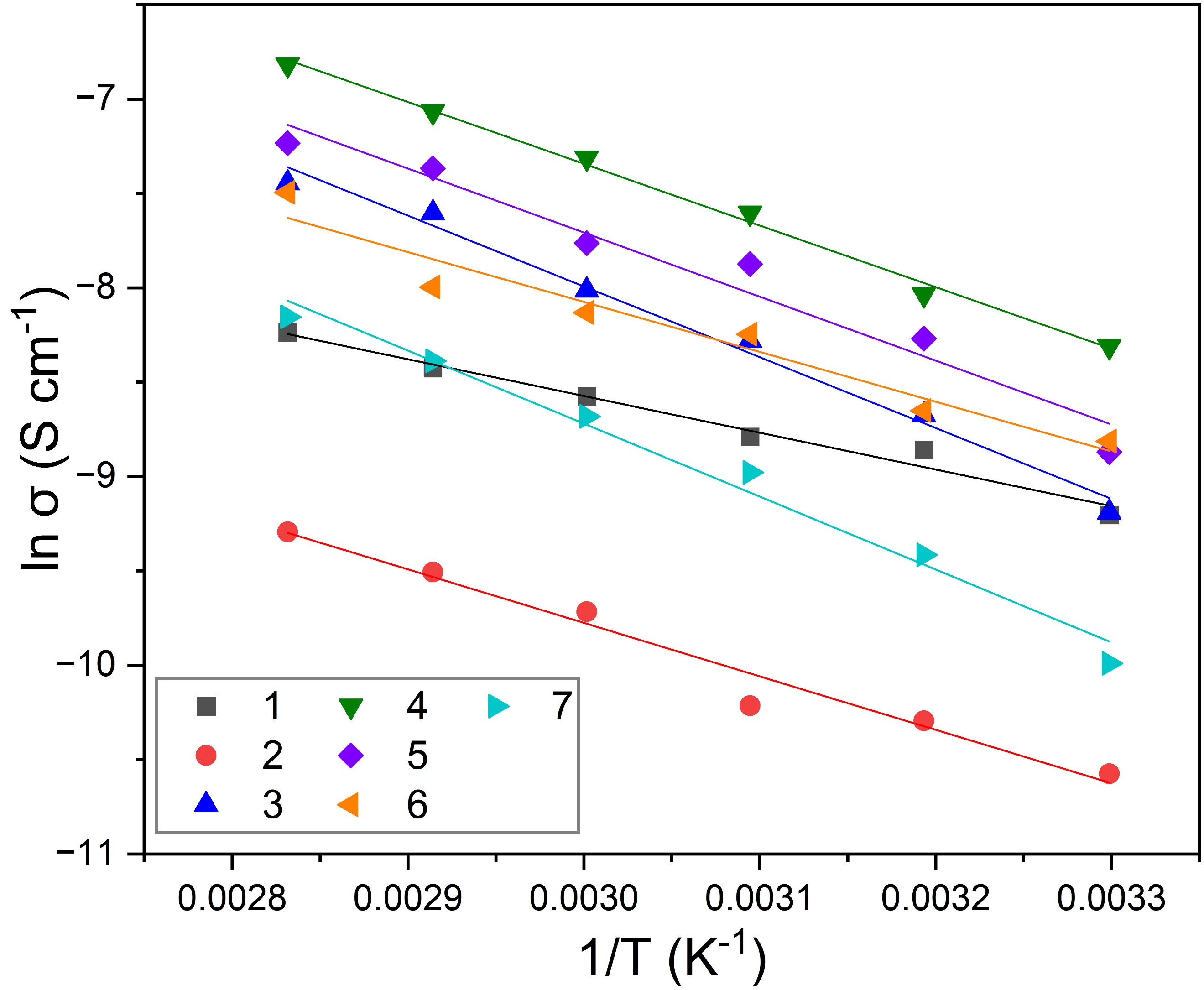


**Fig. S13** Fitted curves of the Arrhenius equation


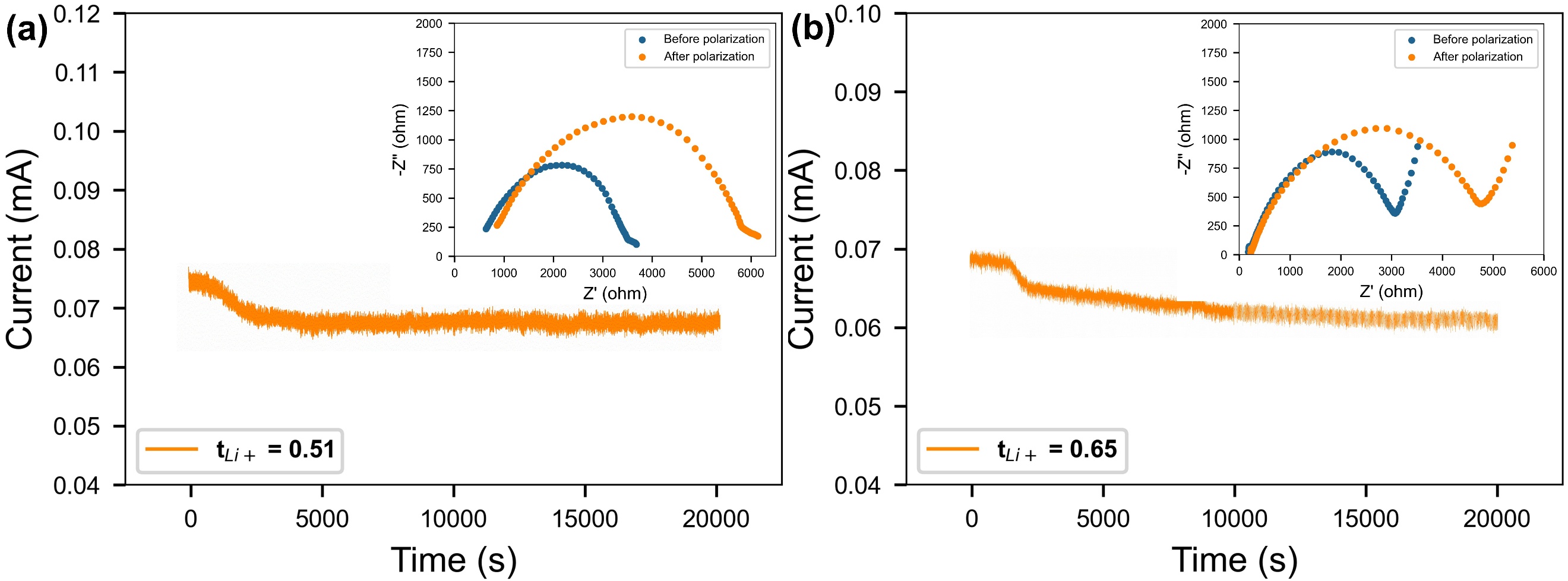


**Fig. S14** Lithium migration numbers of (**a**) FF/ASSPE and (**b**) PVDF-HFP/ASSPE (inset: the impedance before and after polarization)


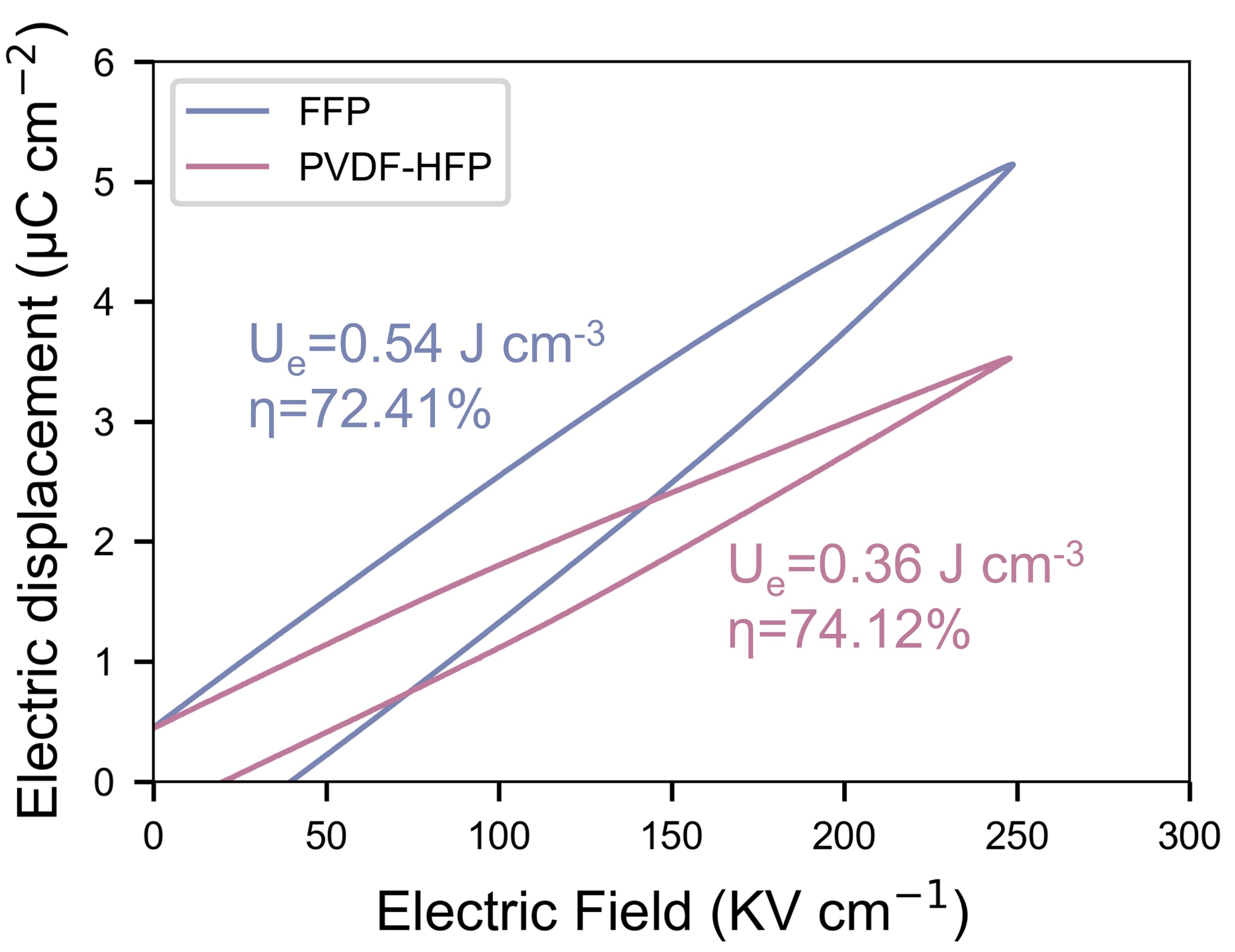


**Fig. S15** D-E loops of FFP and PVDF-HFP


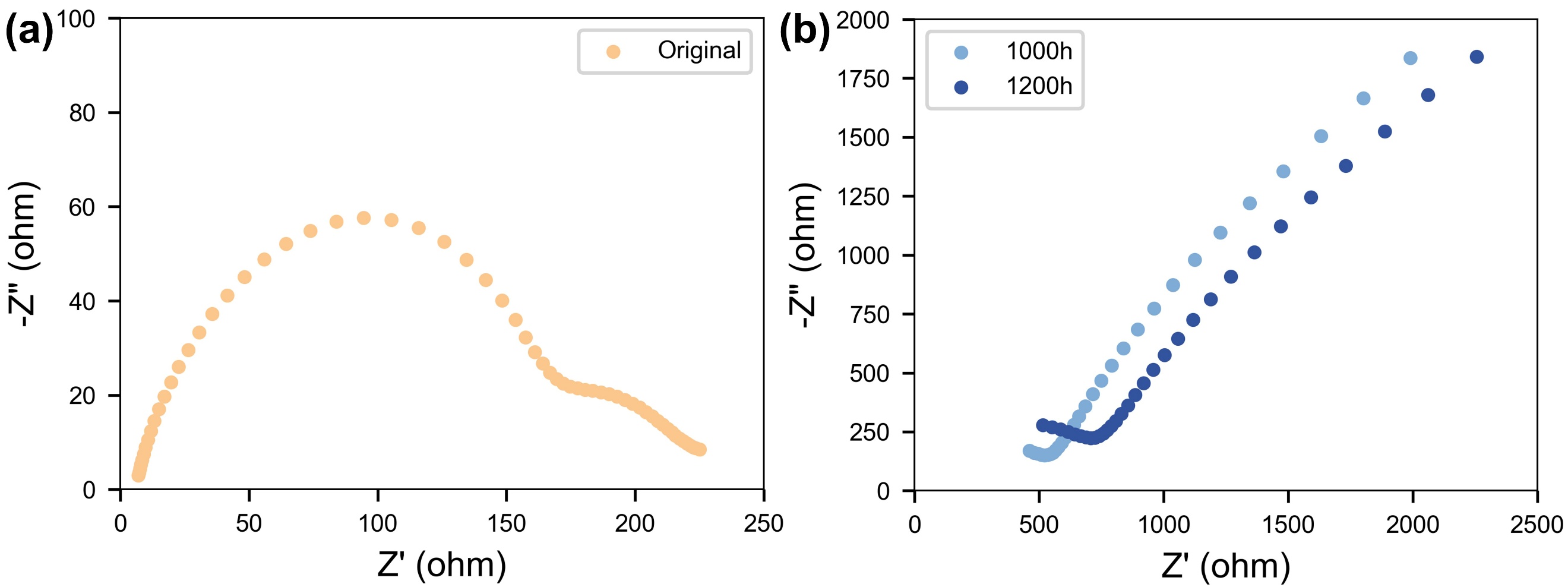


**Fig. S16** EIS plots of (**a**) original Li|FFP/ASSPE|Li and (**b**) Li|FFP/ASSPE|Li after 1000 h and 1200 h cycles at 0.2 mA cm^-2^


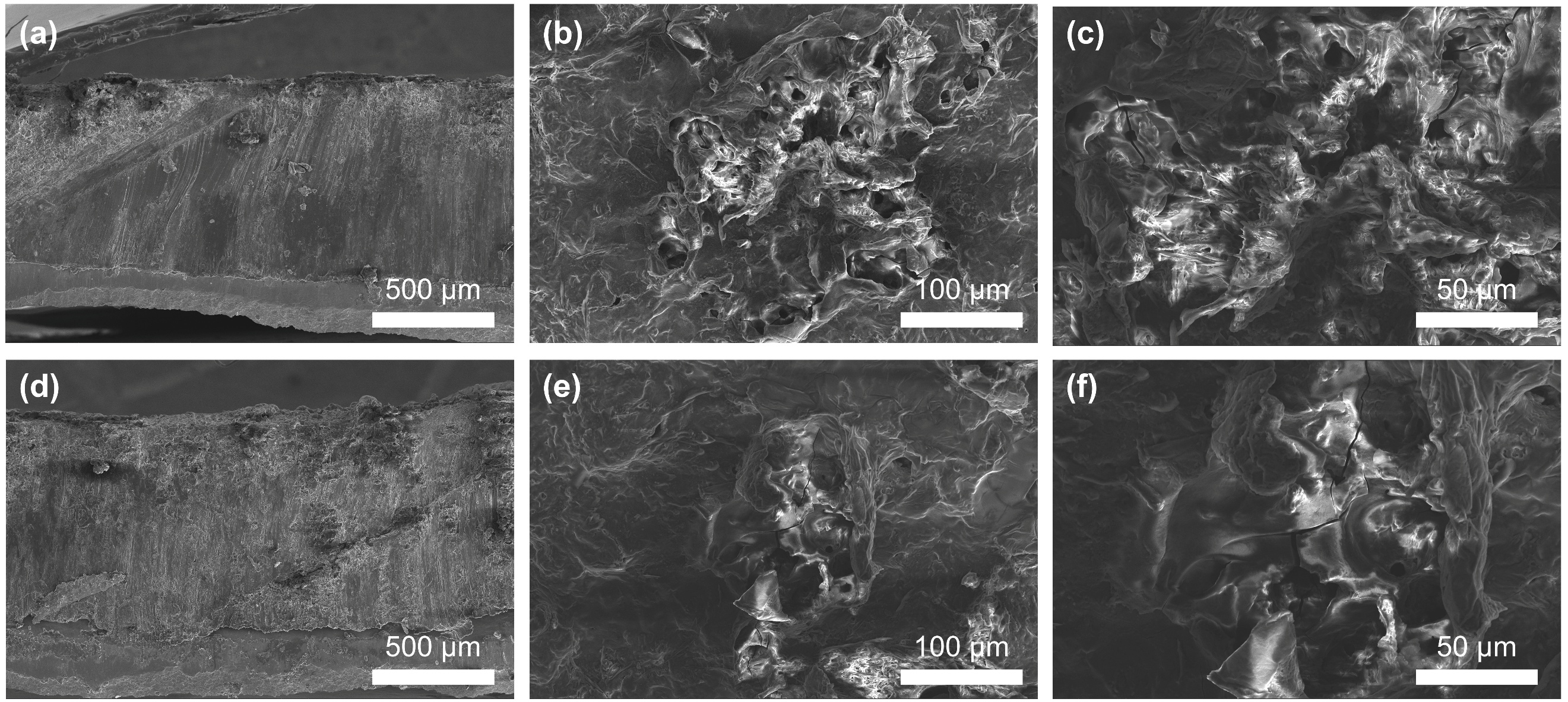


**Fig. S17** (**a**) Cross-section and (**b-c**) surface SEM images of the lithium electrode of Li|PVDF-HFP/ASSPE|Li after 1200 h cycles at 0.1 mA cm^-2^. (**d**) Cross-section and (**e-f**) surface SEM images of the lithium electrode of Li|FF/ASSPE|Li after 1200 h cycles at 0.1 mA cm^-2^


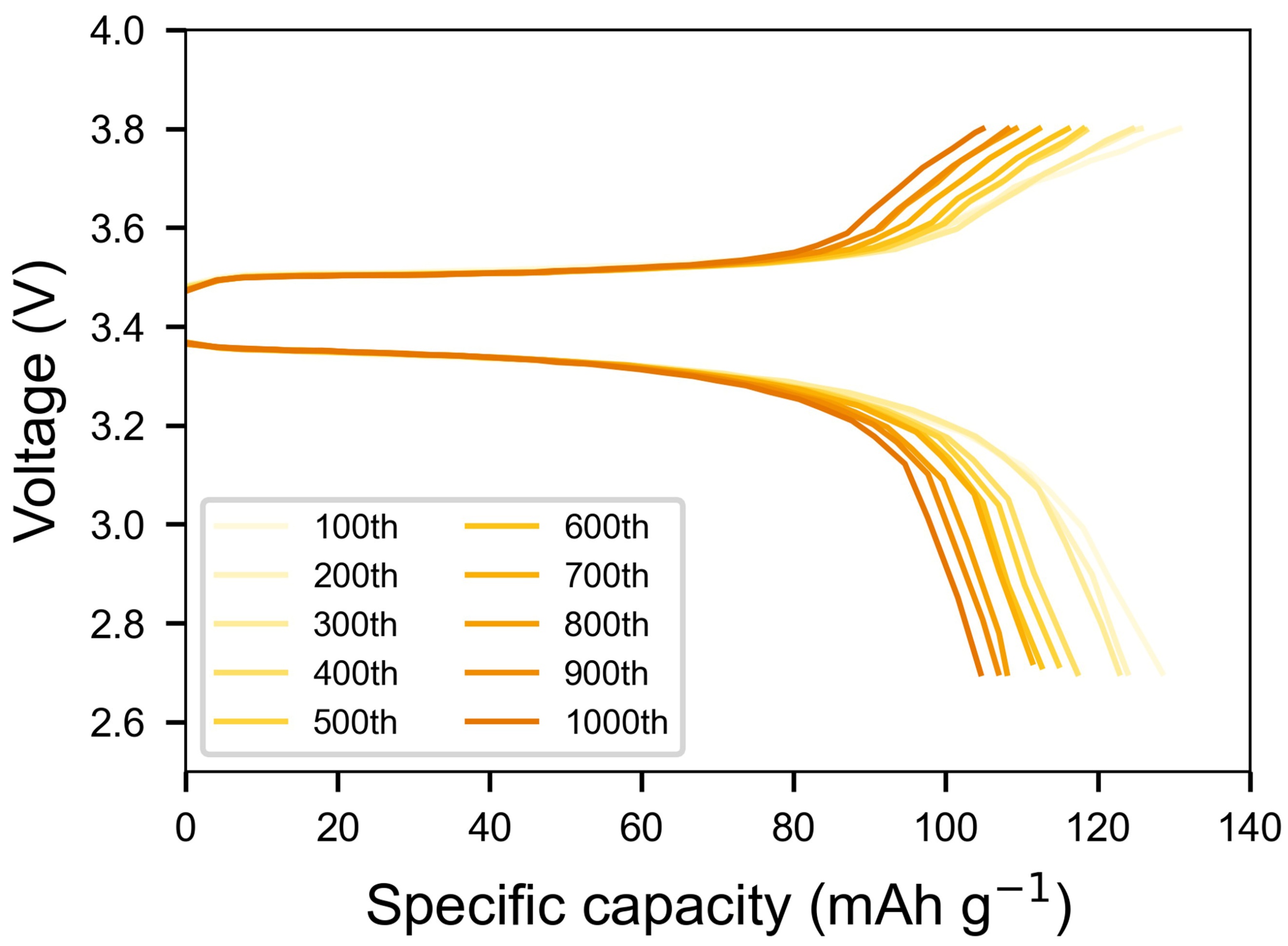


**Fig. S18** Charge-discharge curves of Li|FFP/ASSPE|LFP at 1 C

**
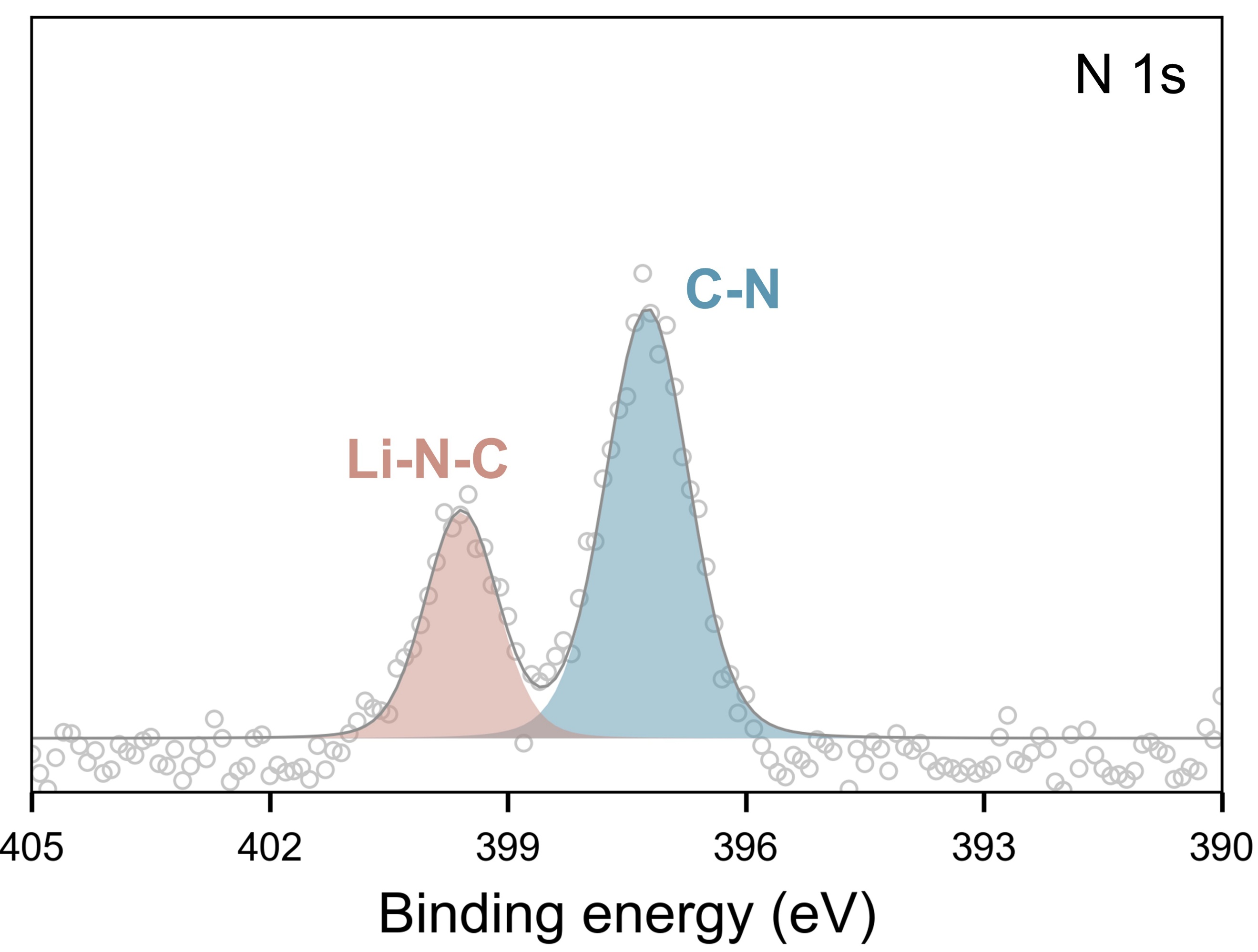
**

**Fig. S19** XPS spectra of lithium electrode of Li|FFP/ASSPE|LFP after 1000 cycles at 1 C


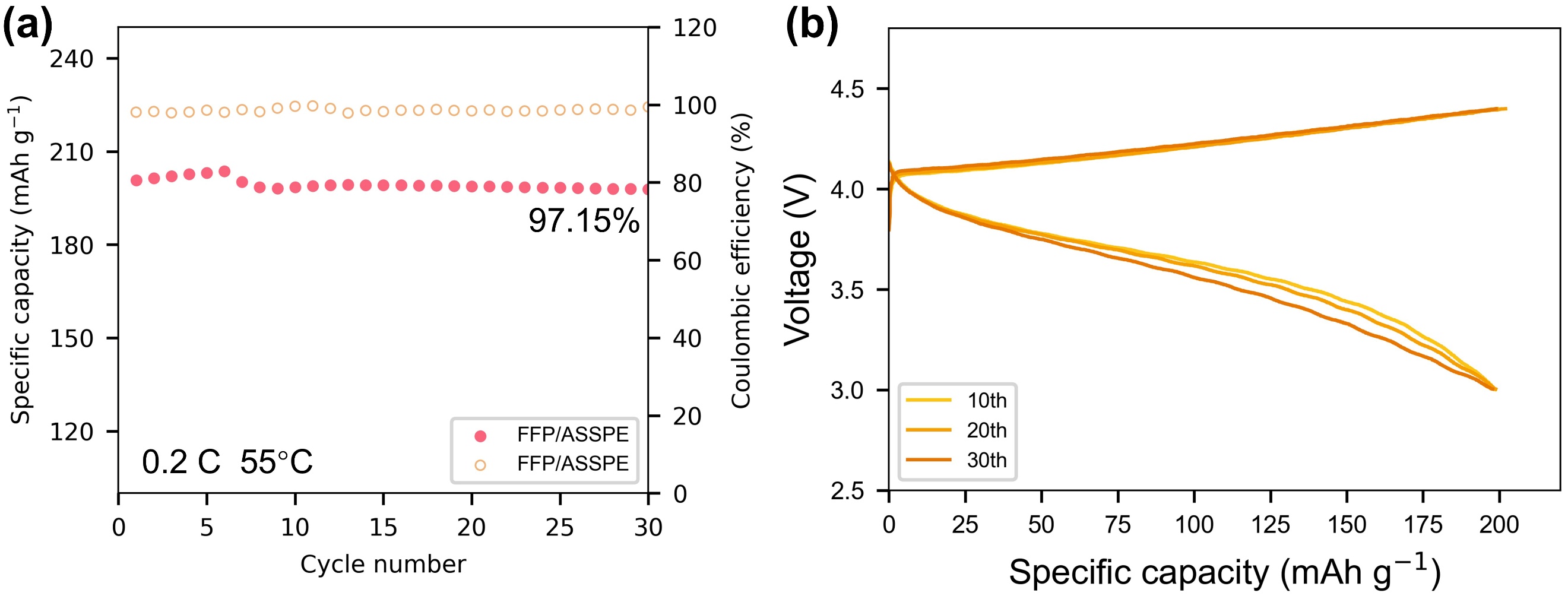


**Fig. S20** (**a**) Cycling performance and (**b**) charge-discharge curves of Li|FFP/ASSPE|LCO at 0.2 C


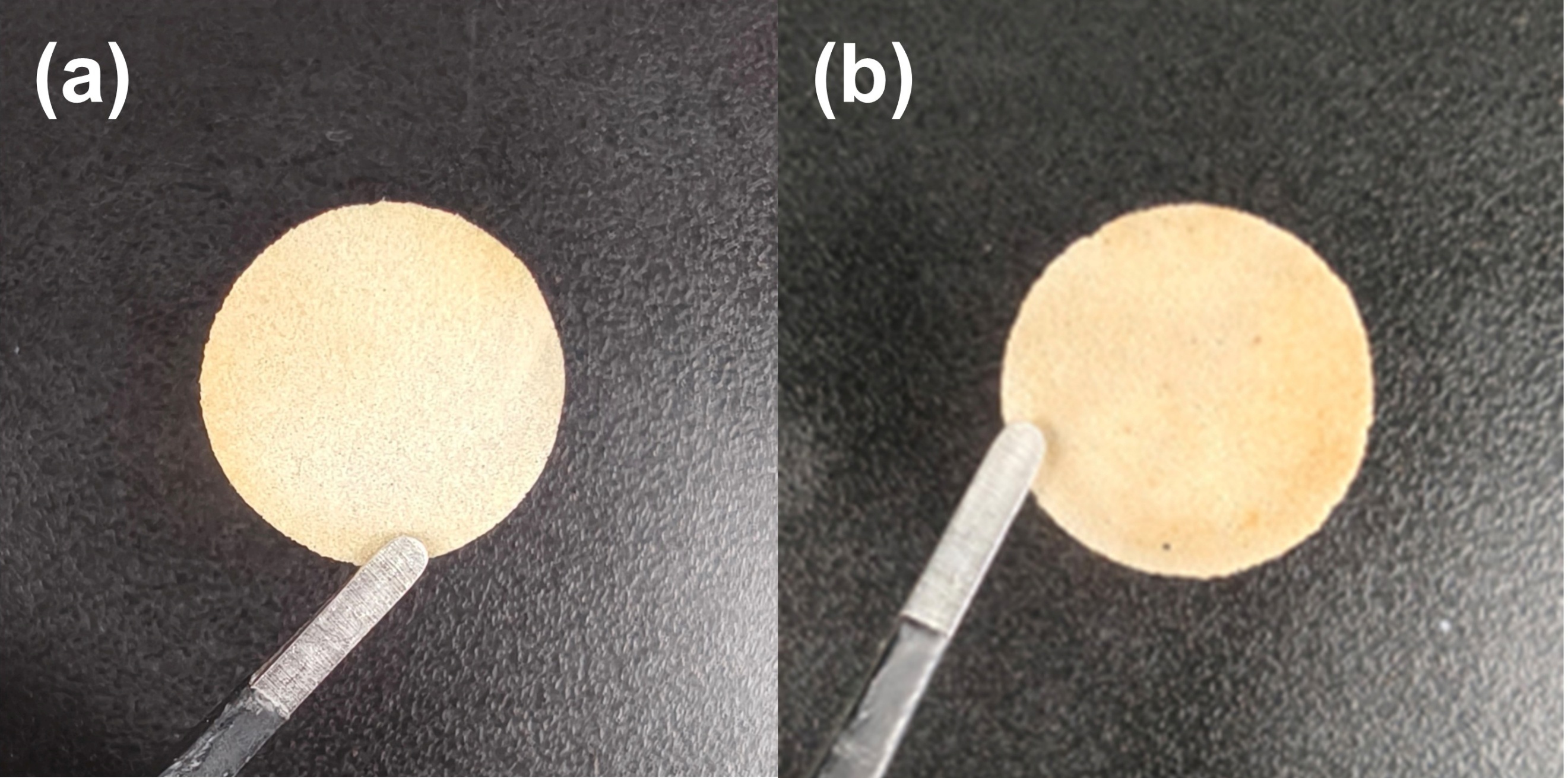


**Fig. S21** Electronic photos of FFP/ASSPE (**a**) before and (**b**) after 120ºC heating


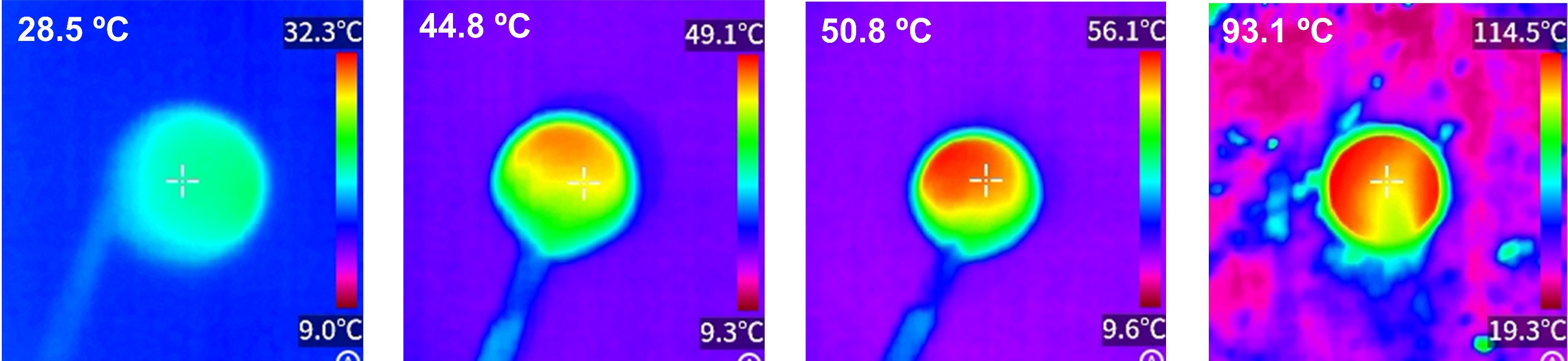


**Fig. S22** Infrared thermogram of FFP/ASSPE at different temperatures

**Table S1** Composite membrane component ratio

| Number | F-nanocellulose/g | PVDF-HFP/g |
| --- | --- | --- |
| 1 | 0.00 | 0.45 |
| 2 | 0.10 | 0.35 |
| 3 | 0.15 | 0.30 |
| 4 | 0.20 | 0.25 |
| 5 | 0.25 | 0.20 |
| 6 | 0.30 | 0.15 |
| 7 | 0.45 | 0.00 |

**Table S2** The activation energies and pre-exponential factors of samples

| Number | *E_a_*/eV | *σ_0_*/S cm^-1^ |
| --- | --- | --- |
| 1 | 0.17 | 6.51 × 10^-2^ |
| 2 | 0.24 | 2.79 × 10^-1^ |
| 3 | 0.32 | 26.12 |
| 4 | 0.28 | 11.74 |
| 5 | 0.29 | 11.71 |
| 6 | 0.23 | 8.62 × 10^-1^ |
| 7 | 0.33 | 17.79 |

**Table S3** The coordination numbers of FF/PVDF-HFP/FFP

| Distance | Li^+^-O in TFSI^-^ | Li^+^-O in F-cellulose | Li^+^-F in F-cellulose | Li^+^-F in PVDF-HFP |
| --- | --- | --- | --- | --- |
| 1Å | 0/0/0 | 0/-/0 | 0/-/0 | -/0/0 |
| 2Å | 1.68/2.32/1.82 | 0.88/-/0.73 | 0/-/0 | -/0/0.08 |
| 3Å | 2.47/3.08/2.54 | 1.46/-/1.07 | 0.15/-/0.08 | -/0/0.53 |
| 4Å | 3.78/4.89/4.02 | 1.60/-/1.14 | 0.84/-/0.39 | -/0.26/1.81 |
| 5Å | 5.62/6.74/5.76 | 2.72/-/2.00 | 2.88/-/1.27 | -/59.66/4.53 |
| 6Å | 7.00/8.31/6.96 | 3.72/-/2.52 | 6.77/-/3.06 | -/117.68/9.01 |
| 7Å | 8.29/9.93/8.08 | 5.71/-/3.69 | 14.37/-/6.76 | -/140.17/16.47 |
| 8Å | 9.48/11.23/8.94 | 8.05/-/4.99 | 26.32/-/12.98 | -/171.66/28.01 |
| 9Å | 10.85/12.73/10.03 | 10.83/-/6.48 | 42.63/-/21.66 | -/196.79/43.56 |
| 10 Å | 12.54/14.54/11.40 | 14.25/-/8.37 | 63.89/-/32.90 | -/229.75/63.35 |

**Table S4** Comparison of the performances of separators

| Separator | *σ*/S cm^-1^ | LFP Capacity retention | Refs. |
| --- | --- | --- | --- |
| Al_2_O_3_@BC | 4.02 × 10^-4^ | 95.4 % (0.2C, 100 cycles) | [S5] |
| PDOL@ZnO/PVDF-HFP | 2.20 × 10^-4^ | 91.3% (0.2C, 600 cycles) | [S6] |
| L@K/C | 1.62 × 10^-4^ | 84.4% (0.2C, 300 cycles) | [S7] |
| PW_12_@UIO66-PP | 7.80 × 10^-4^ | 84.5% (1C, 700 cycles) | [S8] |
| ANF_60_ | 1.50 × 10^-4^ | 80% (0.5C, 240 cycles) | [S9] |
| PPTA/LLZTO | 3.23 × 10^-4^ | 95.6% (0.2C, 1000 cycles) | [S10] |
| PPLT | 1.02 × 10^-4^ | 88% (1C, 700 cycles) | [S11] |
| PVDF/LLZTO | 1.40 × 10^-4^ | 97.5% (1C, 300 cycles) | [S12] |
| FFP | 2.46 × 10^-4^ | 77.5% (1C, 1000 cycles) | This work |

**Supplementary References**

1. M. Ghasemlou, F. Daver, E.P. Ivanova, Y. Habibi, B. Adhikari, Surface modifications of nanocellulose: From synthesis to high-performance nanocomposites. Prog. Polym. Sci. **119**, 101418 (2021). <https://doi.org/10.1016/j.progpolymsci.2021.101418>
2. C. Huang, H. Ji, Y. Yang, B. Guo, L. Luo et al., TEMPO-oxidized bacterial cellulose nanofiber membranes as high-performance separators for lithium-ion batteries. Carbohydr. Polym. **230**, 115570 (2020). <https://doi.org/10.1016/j.carbpol.2019.115570>
3. C. Gao, X. Hu, Y. Huang, X. Ma, *In-situ* solidification POSS-crosslinked polymer electrolytes in multiscale nanocellulose membranes for high-performance all-solid-state lithium batteries. Compos. Part B Eng. **305**, 112736 (2025). <https://doi.org/10.1016/j.compositesb.2025.112736>
4. C. Gao, H. Zhang, X. Hu, X. Ma, *In situ* generating poly ionic liquid composite electrolytes supported by mesoporous silica–modified PP separator enabling stable lithium-ion batteries. Ionics **31**(8), 7773–7783 (2025). <https://doi.org/10.1007/s11581-025-06445-4>
5. C. Cheng, C. Zhao, S. Cai, Y. Tian, R. Lin et al., Functionalizing separator for dual application in liquid and solid-state lithium metal batteries: Achieving stable cycling from − 20 ℃ to 60 ℃. Chem. Eng. J. **508**, 160830 (2025). <https://doi.org/10.1016/j.cej.2025.160830>
6. H. Gao, Y. Zhou, K. Wang, B. Li, S. Wang et al., An *in situ* polymerized solid-state electrolyte for uniform lithium deposition *via* the piezoelectric effects. Adv. Energy Mater. **15**(28), 2501379 (2025). <https://doi.org/10.1002/aenm.202501379>
7. W. Sun, J. Zhang, M. Xie, D. Lu, Z. Zhao et al., Ultrathin aramid/COF heterolayered membrane for solid-state Li-metal batteries. Nano Lett. **20**(11), 8120–8126 (2020). <https://doi.org/10.1021/acs.nanolett.0c03133>
8. Y. Liu, T. Hou, W. Zhang, B. Gou, F. Li et al., Anion-repulsive polyoxometalate@MOF-modified separators for dendrite-free and high-rate lithium batteries. Interdiscip. Mater. **4**(1), 190–200 (2025). <https://doi.org/10.1002/idm2.12225>
9. S. Liu, S. Cheng, C. Huang, J. Han, J. Xie et al., Nanoporous aramid nanofiber separators with high modulus and thermal stability for safe lithium-ion batteries. Small **20**(49), 2404639 (2024). <https://doi.org/10.1002/smll.202404639>
10. Y. Mao, W. Sun, Y. Qiao, X. Liu, C. Xu et al., A high strength hybrid separator with fast ionic conductor for dendrite-free lithium metal batteries. Chem. Eng. J. **416**, 129119 (2021). <https://doi.org/10.1016/j.cej.2021.129119>
11. P. Liu, L. Zhong, S. Wang, S. Huang, D. Han et al., Ultra-long-life and ultrathin quasi-solid electrolytes fabricated by solvent-free technology for safe lithium metal batteries. Energy Storage Mater. **58**, 132–141 (2023). <https://doi.org/10.1016/j.ensm.2023.03.012>
12. H. Huo, X. Li, Y. Chen, J. Liang, S. Deng et al., Bifunctional composite separator with a solid-state-battery strategy for dendrite-free lithium metal batteries. Energy Storage Mater. **29**, 361–366 (2020). <https://doi.org/10.1016/j.ensm.2019.12.022>
